# Initial prejudices create cross-generational intergroup mistrust

**DOI:** 10.1371/journal.pone.0194871

**Published:** 2018-04-25

**Authors:** Eric Luis Uhlmann, Aleksey Korniychuk, Tomasz Obloj

**Affiliations:** 1 Organisational Behaviour Area, INSEAD, Singapore, Singapore; 2 Department of Strategic Management and Globalization, Copenhagen Business School, Kilevej 14, DK, Frederiksberg, Denmark; 3 Strategy and Business Policy, HEC Paris, Jouy-en-Josas, France; Fordham University, UNITED STATES

## Abstract

The present investigation modeled the emergence and persistence of intergroup bias and discrimination in artificial societies. Initial unfair prejudices held by members of a dominant group elicit confirmatory behavior (diminished cooperation) from members of a subordinate group via a self-fulfilling prophecy. Further, when individual learning is tempered by conformity to peers, inaccurate beliefs about the stigmatized subordinate group persist long-term. Even completely replacing dominant group members with enlightened individuals through generational change is inadequate to break the cycle of intergroup distrust and non-collaboration. The longer the enlightenment of a society is delayed, the more intergroup trust is irretrievably lost.

## Introduction

Among the most intractable problems facing human societies across the world is that of group based discrimination and inequality. Social stratification is a human universal [1], and the correlate of the emergence of an elite is the development of subordinate groups characterized by social marginalization and a lack of economic resources [2, 3, 4]. Members of socially stigmatized and negatively stereotyped ethnic, religious, and cultural groups are chronically overrepresented among the most disadvantaged members of a society [1]. Such group-based inequality persists into comparatively more enlightened times, in which the virulent prejudices of the past, if certainly far from absent, have at least abated significantly [5].

The simplest explanation for the resilience of group-based inequality across generations of many societies is that it constitutes a tragic residue of the blatant discrimination of the past [6, 7]. The historical record is replete with cases in which a dominant group brutally oppressed subordinate groups through conquest, colonialism, slavery, and/or forced segregation, leading to savage inequalities in objective resources. Under conditions of inherited wealth, initially disadvantaged groups will continue to suffer deficits in economic resources, educational attainment, and human capital long into the future.

Although objective and persisting gaps in resources are certainly one of the fundamental causes of the problem, the present research highlights the role of subjective, psychological variables in creating patterns of group-based mistrust, marginalization, and inequality. Through the analysis of interactions in artificial societies, we show that prejudice toward members of a subordinate group in the early stages of a society elicits confirmatory behavior (reduced cooperativeness) via a self-fulfilling prophecy, creating a sustained cycle of intergroup mistrust and non-collaboration. The terms *prejudice* and *stigma* are used here interchangeably to refer to inaccurate and negative beliefs about a subordinate group’s worth as collaborators [8, 9, 10]. We assume that some prejudice exists at the very beginning of intergroup interactions. *Discrimination* is used to refer to the behavior of choosing not to collaborate with a member of a subordinate group based on prejudiced beliefs about them. This is of course a simplification of real-world intergroup interactions, which involve multifaceted and in some cases contradictory stereotypes, attitudes, and norms related to other social groups. However, in line with previous work using agent based modeling to examine intergroup processes [11] we use cooperation (vs. defection) and relevant beliefs about the trustworthiness of the counterpart as our operationalizations of intergroup behaviors and attitudes, respectively.

In our simulations, societal prejudices and discrimination give rise to a self-fulfilling prophecy [12, 13], as discriminated-against subordinate groups develop response patterns they did not initially possess, learning to mistrust members of the dominant group [14, 15]. Distinct from the recent work on the origin of social biases [11, 16], our analysis focuses on the destructive consequences of the initial prejudiced treatment of the subordinate group, at the same time pointing to the roots of the troubling persistence of such unfair beliefs. To this effect we demonstrate that despite some changes in the actual cooperativeness of members of the subordinate group in response to initial prejudices against them, beliefs about the subordinate group continue to be harmfully inaccurate long into societies’ development, and under some plausible conditions, never converge with actual behaviors. Thus, although unfair discrimination leads stigmatized groups to develop the very lack of cooperativeness they were presupposed to have, they do not become as uncooperative as members of the dominant group believe they are.

Perhaps most dramatically, we find that when initial generations of the dominant group are prejudiced and some degree of conformity to peers exists, even a *complete replacement* of prejudiced dominant group members with non-prejudiced individuals through generational change fails to end the cycle of intergroup mistrust and non-collaboration [17]. Although ameliorating the effects of past discrimination always poses a challenge, the longer the enlightenment of the dominant group is delayed, the more intergroup relations are irreparably damaged.

## Method

**Fig 1:**
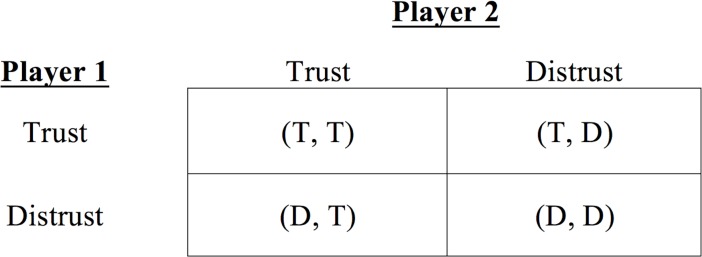
Coordination game structure. In this game there are two pure strategy Nash equilibria: (T, T) and (D, D). We Pareto rank equilibria and assume that (T, T) is strictly superior to (D, D) for both players. Both equilibria dominate outcomes that do not satisfy the mutual best-response criterion: (T, D) and (D, T). We assume that players cannot credibly reveal their intentions prior to the interaction and therefore must act on their beliefs about others. As beliefs are an imperfect representation of the state of the world, coordination failures arise.

We conceive of each individual social interaction as a coordination game, where players have no incentive to cheat but must choose strategies based on their priors about others [18, 19, 11, 20, 21]. Each player has two generic strategies: collaborate or not. Consistent with the studied phenomenon, the two existing Nash equilibria are Pareto-ordered and we refer to the superior equilibrium as *collaboration* or *trust*, while calling the inferior one *noncollaboration* or *distrust*. Fig 1 illustrates the structure of the game. The intuition is that each player gains more if they collaborate and less if they do not. This is consistent with the simple idea that trust is an important determinant of the social welfare [22, 23].

We think of the game as involving two stages: (i) formation of a pure-strategy belief, and (ii) acting on this belief. Stage (i) is subject to learning and initial biases [14] as agents make their own, idiosyncratic assumptions, seeing either trustworthiness in their counterparts or the lack thereof. Stage (ii) represents the choice of strategy.

From the structure of the game (see Fig 1) it follows that players will opt for a pure strategy that reflects their beliefs about others. In simple terms, our agents trust those they perceive as high quality collaborators and do not invest in interactions with those they see as low quality collaborators. Note that we do not assume any specific payoffs to strategies. Rather we consider only behavioral aspects of a game where players decide to collaborate or not based on their preconceived expectation about the probable choice of the other player. Such a conception of social interactions allows for a clear definition of human bias. If, given some criterion, player *i* is less likely to see a quality collaborator in player *j* than the latter in fact tends to be, player *i* can be said to be biased along this criterion.

Building on the large body of previous formal work with a modified approach designed to capture intergroup dynamics [18, 24, 25], we assume that players can discriminate between groups rather than just individuals [26]. At any moment in time a member of group *I* is equally likely to collaborate with (i.e., see a desirable collaborator in) any member of group *J*. Interactions are selected at random from all possible dyadic interactions, meaning that the probability of interacting with any member of group *J* (as opposed to any member of group *I*) is proportional to the relative size of the two groups. Unless otherwise indicated, we assume |*I*| = 80 and |*J*| = 20. We present results for alternative relative group sizes (see S8–S12 Figs).

In our model, the individual *i*'s (*i* ∈ *I*) probability (*ρ*_*i*:*J*,*t*_) to collaborate with any individual *j* in group *J* in period *t* is subject to the following learning process [19, 20]:

$$\rho_{i:J,t}=\alpha_{i}\cdot s_{j,t-1}+\left(1-\alpha_{i}\right)\cdot \rho_{i:J,t-1}.$$

Where *s*_*j*,*t*_ is the strategy chosen by the player from group *J* in a given interaction, such that *s*_*j*,*t*_ is 1, when she collaborates and 0 otherwise, and *α*_*i*_ ∈ [0,1] is the learning parameter. Note that in our simulations all dyadic-level interactions are random. In other words, any member of group *I* is equally likely to interact with any other individual, irrespective of whether this individual belongs to group *I* or to group *J*. Further, an agent updates her priors about the entire group. This learning process implicitly captures the concept of indirect reciprocity in that an individual *i* changes her priors to reciprocate what she experienced in the last interaction with a certain group [25].

We consider two groups, *I*, dominant and *J*, subordinate, where initially, the former wrongly sees the latter as low quality collaborators, i.e. ∀*i* ∈ *I*, *j* ∈ *J*, *E*(*ρ*_*i*:*J*,*0*_) *< E*(*ρ*_*j*:*I*,*0*_). At the individual level we allow for variation in beliefs [27] so that systematic discrimination occurs only at the group level. Specifically, we consider a population, where *ρ*_*j*:*I*,*0*_ ~ *U*(0,1) and *ρ*_*i*:*J*,*0*_ ~ *U*(0,*θ*), *θ* < 1 (our findings are qualitatively robust to a wide spectrum of alternative specifications and distributions of priors, see S1 and S2 Files, S1–S12 Figs). Such asymmetry captures the idea of prejudice and social stigma in that members of the dominant group on average unfairly believe that members of the subordinate group are lower quality collaborators than they actually are.

In sum, our model portrays a society in which people form idiosyncratic beliefs about members of other groups and act on these beliefs in their decision making. At the outset, members of this society differ in their propensity to trust other people and as such vary in their willingness to collaborate. Our agents are also liable to flaws of human judgment. At the group level, some members unjustly see bad qualities in those who differ from them. These beliefs, however, change as individuals interact and learn about each other. With the help of this model we trace transformations in intergroup relations that occur in our society over time.

The random and learnt aspects of the model make formal analysis prohibitively difficult. Accordingly, to analyze social processes that span eras of history we use agent-based modeling [18, 24, 28, 26, 29, 30]. The code for this model was written in C++ (see S1 File). Below we describe our observations from the modeled social process.

## Results and discussion

**Fig 2:**
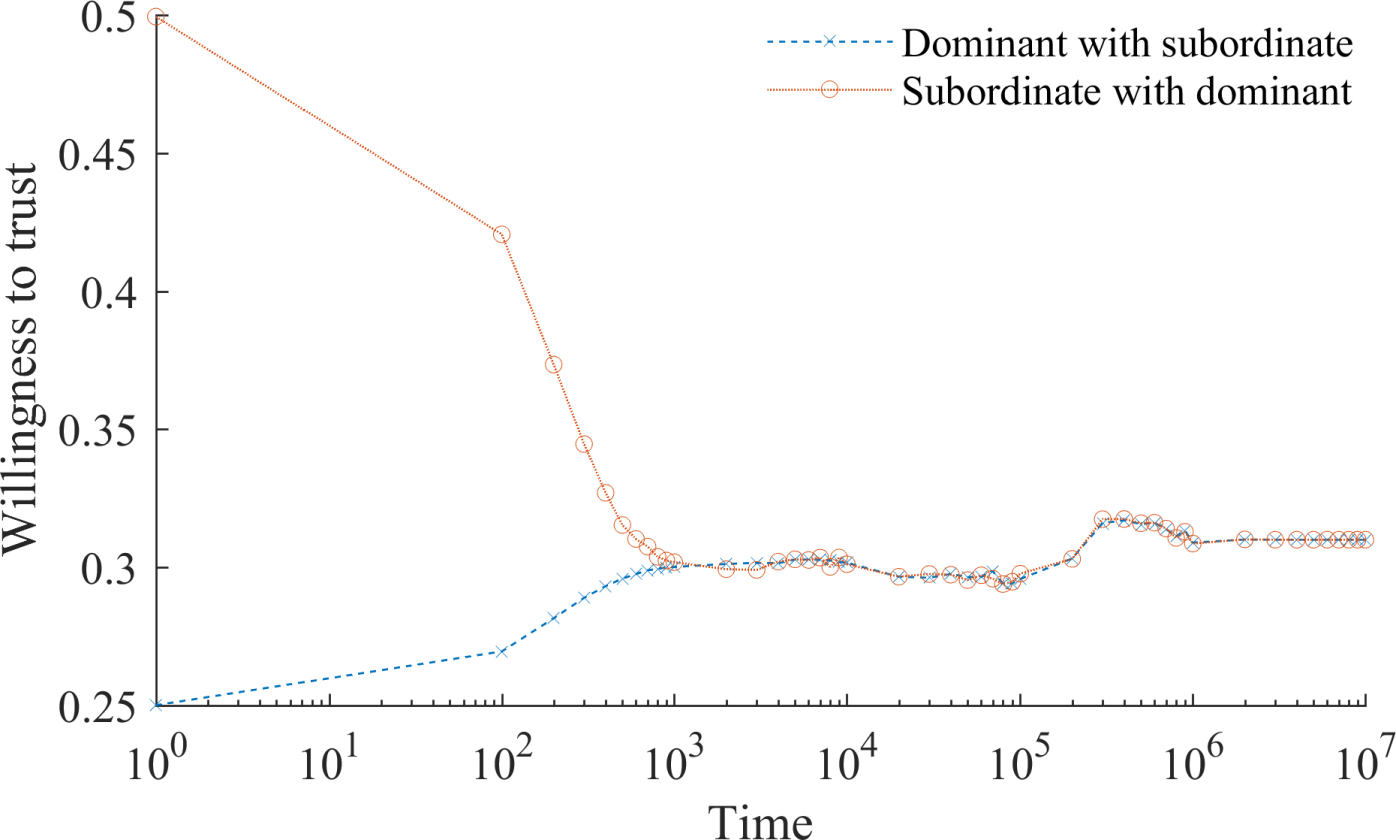
The self-fulfilling prophecy of social stigma. Fig 2 shows the dynamics of willingness to trust when learning speed, *α* is constant (without loss of generality set equal to 0.25). Over time the stigmatized subordinate group becomes less naïve and learns not to trust the prejudiced dominant group. Similarly, members of the dominant group learn that subordinate group members are more trustworthy than they initially thought. Convergence in beliefs occurs at ~0.3 willingness to trust.

Interaction and learning drive social mentality. Naturally, when *α*_*i*_ is 0 and no learning occurs, initial prejudices persist indefinitely. When, in contrast, *α*_*i*_ is constant and greater than 0 any inaccurate belief about the subordinate group disappears in the limit. The flipside of learning is that members of the stigmatized group learn not to naïvely collaborate and gradually suppress their readiness to trust in the face of biased treatment from the dominant group (see Fig 2). Unjust social stigma thus provokes a symmetric behavioral response from the stigmatized, giving rise to a vicious circle of distrust. This creates a self-fulfilling prophecy, as members of the subordinate group who were initially willing to collaborate with dominant group members learn not to invest in collaboration instead. Distrust in turn increases the frequency with which individuals enter inferior equilibrium. For a large class of problems, where mutual trust is a key condition for value creation, this implies a comparative wealth disadvantage for the stigmatized subordinate group.

Counterintuitively, the time that it takes for this negative intergroup dynamic to stabilize decreases with *α*_i_ (see S1 Fig). Although a higher rate of learning might be presumed to cause dominant group members to reject negative beliefs about the subordinate group, it actually leads biased beliefs to become self-fulfilling more quickly. As a result, subordinate group members rapidly learn that dominant group members are biased against them and hence really do become reluctant to invest in collaborations.

The speed of learning itself is a product of factors that are largely exogenous to the process of intergroup interaction, with the important exception of social conformity. We model social conformity by allowing the extent to which one learns from experience to vary with the deviation of her belief from those of other ingroup members. Given that members with views different than the norm tend to be more likely to adjust their beliefs in response to new information—an effect demonstrated in the context of U.S. racial attitudes and norms [31, 32]—for every individual at any point in time *t*, learning speed *α*_*i*,*t*_ is set proportional to the deviance of one’s belief from the group average. The less representative a given member’s beliefs are of those in her group (i.e., the greater the distance between her prior and the group norm), the more likely she is to change her views in response to personal experiences with outgroup members. This characterization captures the idea, supported by experimental research, that behavior is comparatively “sticky” around the group norm and malleable away from it [31, 32].

Specifically, at any moment in time, *α*_*i*,*t*_ is the equivalent of |*ρ*_*i*:*J*,*t*_− *ρ*_*I*:*J*,*t*_|, where *ρ*_*I*:*J*,*t*_ characterizes the group *I*’s norm of collaboration with members of group *J*, and *ρ*_*i*:*J*,*t*_ is individual *i*'s (*i* is a member of group *I*) willingness to collaborate with members of group *J* (similarly defined for *j* and *I*). Our analysis relies on a careful definition of the group norm. Drawing on research examining closeness in social networks [33], we measure the norm as a group-wide weighted average willingness to trust, where weights originate from all pairwise comparisons of priors among the members and are inversely proportional to the average deviance of one’s belief from those of peers.

Formally, for each individual *i*, we calculate the total distance *d*_*i*,*t*_ in beliefs from all group members, such that $d_{i,t}=\sum_{i,k\in I}\left|\rho_{i:J,t}-\rho_{k:J,t}\right|$. We then weight individual beliefs by our distance measure in calculating the group norm such that:

$$\rho_{I:J,t}=\sum_{i\in I}\frac{\rho_{i:J,t}\cdot d_{i,t}^{-1}}{\sum_{i\in I}d_{i,t}^{-1}}.$$

Our main results are qualitatively robust to alternative measures of the group norm where we weight individuals’ beliefs by the absolute distance from the arithmetic group mean or median.

**Fig 3:**
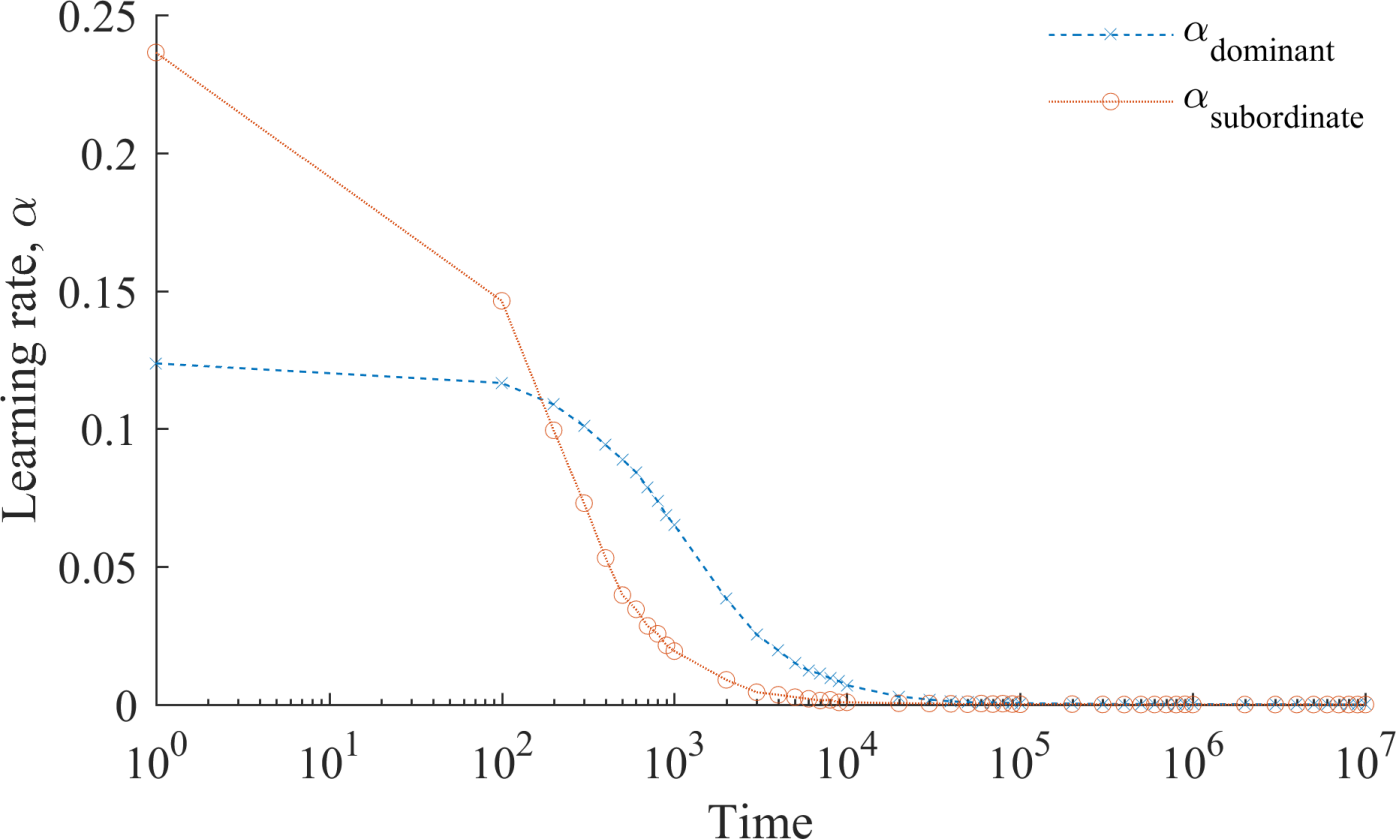
Learning rate in the presence of social conformity. Fig 3 shows the dynamics of the learning speed when individuals are subject to social conformity. Average speed of learning in a group is defined as a simple average of learning rates among all members of this group. Note that the dynamics of the learning rate is key to understanding our analyses. Over time individuals become less and less likely to learn from their experience and increasingly rely on their prior beliefs, which gradually approach the group’s norm. The initial difference between *α*_dominant_ and *α*_subordinate_ is due to the greater variance in beliefs within the subordinate group (cf. S2 Fig). Members of the dominant group preserve a higher learning rate for longer (recall that the learning rate is a function of an individual belief relative to that of the group; greater variance in beliefs, therefore, corresponds to a higher learning rate), because they are more numerous and therefore their group opinion is less sensitive to individual updating of beliefs.

**Fig 4:**
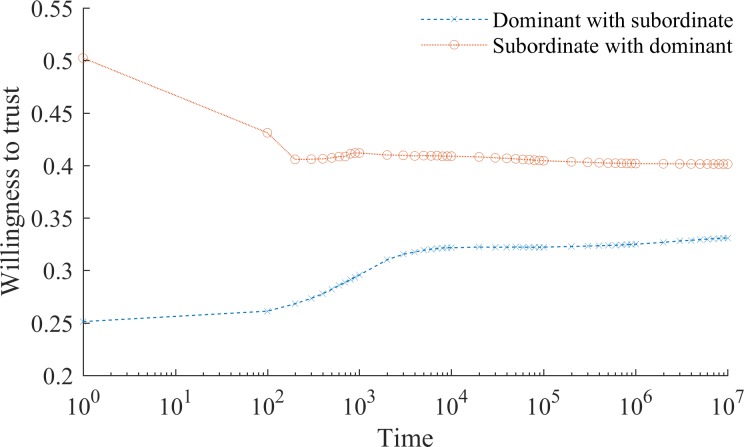
Persistence of stigma. The graph shows the dynamics of intergroup collaboration in the presence of social conformity. The horizontal axis shows time in logarithmic scale. The vertical axis is the average willingness to collaborate with the members of another group (here and below *θ* = 0.5). To offset the effects of random noise the same (randomly seeded) routine was run 100 times. The main observation is that the dynamics of the willingness to collaborate with others largely stabilize for both the subordinate and dominant group before the convergence occurs. At the end of the simulation (*t* = 10^7^), although members of the subordinate group are ready to see quality partners in dominant group members in ~40 percent of intergroup interactions, members of the dominant group respond with equal affinity in only ~34 percent of cases.

In the presence of social conformity *α*_*i*,*t*_ is a decreasing function of accumulated social interactions (see Fig 3). Fig 4 demonstrates that for intergroup collaboration this means that the emergence of a discriminated-against subordinate group is accompanied by a lasting and unjustified stigma against them. Over time, members of the subordinate group become less and less willing to collaborate with the prejudiced dominant group, yet they do not become as unwilling to collaborate as members of the dominant group believe they are. Consider that if *α*_*i*_ → 0, then *ρ*_*i*:*J*,*t*_ → *ρ*_*i*:*J*,*t—1*_. That is, over time individuals become gradually less ready to adjust their beliefs about members of other groups. Sadly, this happens before stigma disappears. The reason for this is that learning about other group happens at the individual level (i.e. *α*_*i*_ is individual specific) whereas convergence to the belief shared by one’s group is in part a group-level phenomenon. Specifically, any cross-group interaction affects the average belief, and therefore *α*_*i*_, but only an interaction that involves a given individual affects his or her subjective probability to collaborate *ρ*_.:.,*t*_. In a stabilized society, therefore, we can expect that alienated subordinate groups coexist with prejudiced dominant groups, who even eras later would still discriminate against those who are not like them.

While there is no direct mapping of model time on to real-world time, two aspects of our analyses allow us to conclude that the reported effects persist in the long run. First, the observed time interval. Consider that in our simulated populations we have 100 individuals and 10^7^ interactions. If every individual has one interaction involving the choice to collaborate or defect with a random person per day, i.e. a total of 50 interactions in the population per day, our modeled time interval translates into over 500 years. Greater frequency of interactions naturally results in a more compressed correspondence with real-world time intervals.

**Fig 5:**
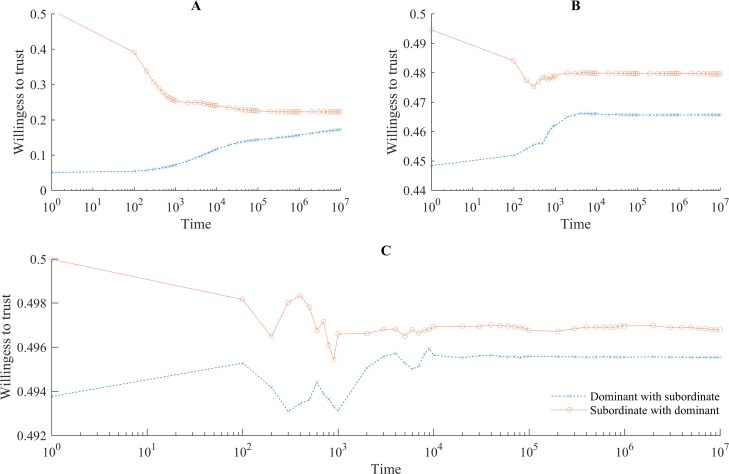
Magnitude of initial prejudice against subordinate group. Fig 5 shows how the dynamics of intergroup collaboration are affected by the initial magnitude of stigma. For panels A through C the subordinate group’s average initial willingness to collaborate is fixed at 0.5, i.e. *ρ*_*j*:*I*,*0*_ ~ *U*(0,1). The distribution for dominant group members’ initial beliefs, however, changes as follows: A) *ρ*_*i*:*J*,*0*_ ~ *U*(0,0.1), B) *ρ*_*i*:*J*,*0*_ ~ *U*(0,0.9), and C) *ρ*_*i*:*J*,*0*_ ~ *U*(0,0.99). Panel C shows that even when initial unfair prejudices are comparatively weak, in the presence of social conformity stigma nonetheless tends to persist.

**Fig 6:**
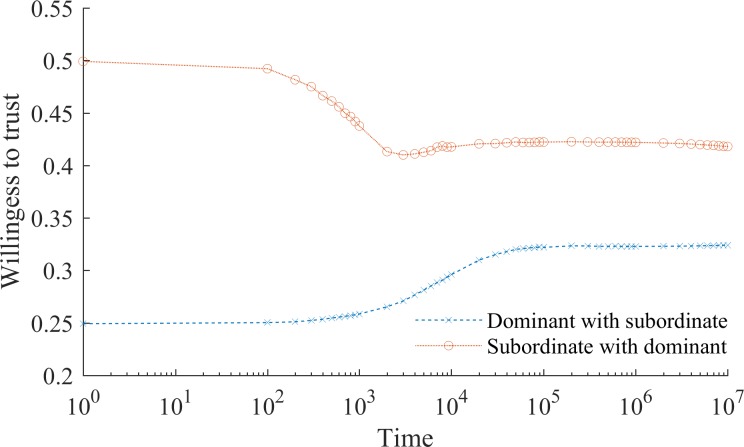
Large population: Base case. Fig 6 shows how the willingness to collaborate with outgroup members in a larger population (i.e. 1000 agents) changes over time. As with a small population, stigma (unfairly negative beliefs about the subordinate group on the part of members of the dominant group) persists. Increasing the size of the population has no qualitative effects on the main observations (see Fig 1).

An important property of these regularities is that even a trivial bias in initial beliefs on the part of the dominant group can result in a lasting social stigma against the subordinate group. Fig 5 shows that the emergence and persistence of a stigmatized subgroup is robust to different degrees of the asymmetry in trust at the onset of cross-group interaction. The effects also hold for larger populations, as seen in Fig 6 for a population of 1000 individuals.

We next ask the question of whether it is possible to break the cycle of intergroup mistrust and non-collaboration by changing the prejudiced beliefs of the dominant group. When conformity to normative beliefs diminishes the ability to learn from individual experiences with outgroup members (*α*_*i*,*t*_) the time to reverse the negative consequences of initial stigma and discrimination for the behavior of members of the subordinate group is, if not infinite, dramatically larger than the time required for such negative behavioral consequences to emerge.

**Fig 7:**
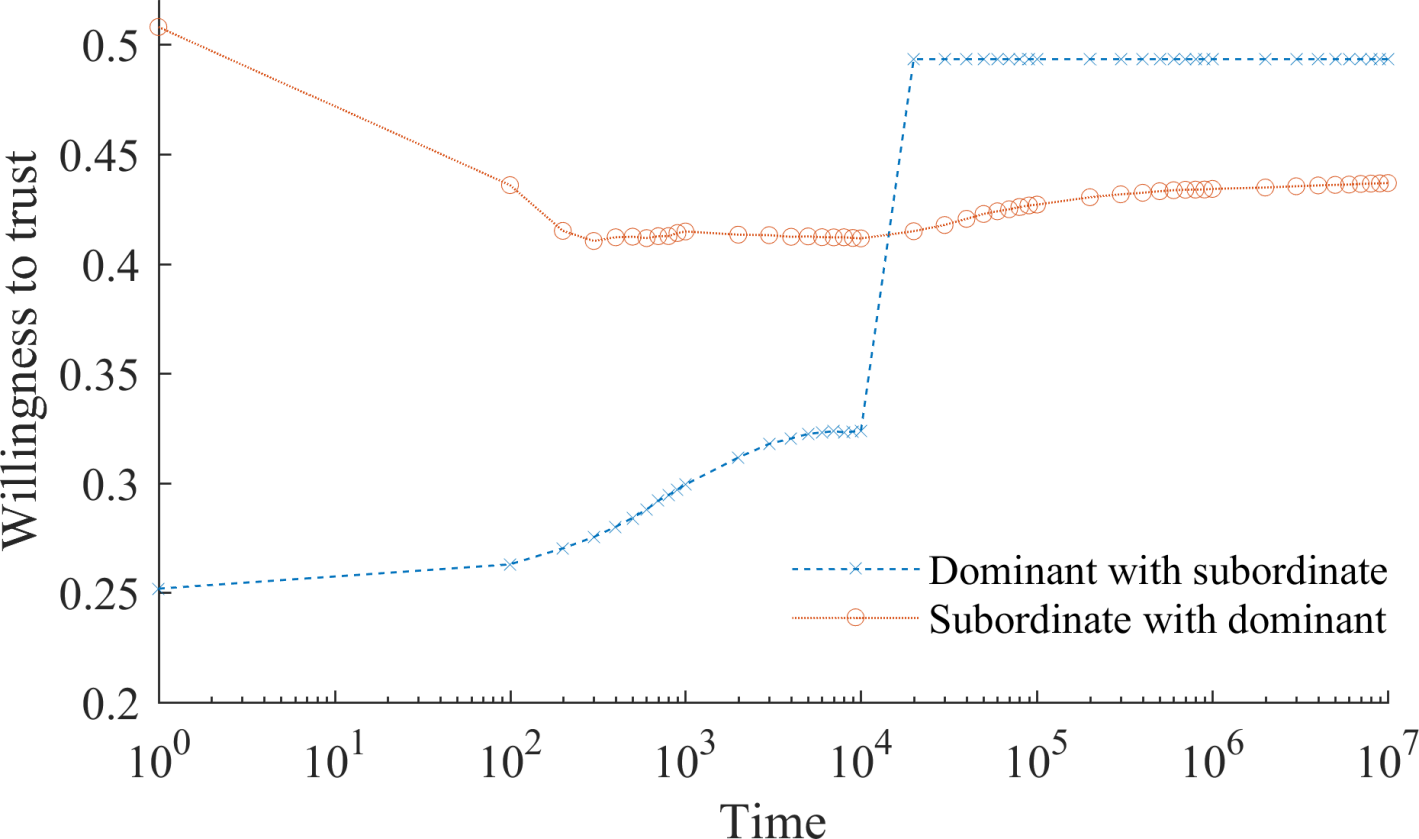
Enlightenment of the dominant group. The horizontal axis depicts time in logarithmic scale. It is assumed that for *t* ≥ 10^4^ every period all members of the dominant group are replaced with perfectly enlightened individuals, who see no difference between dominant and subordinate group members and treat them the same. Note that we consider the case of long-term enlightenment, where dominant group members are prepared to continue to collaborate despite a learned unwillingness on the part of the previously stigmatized group. While at the early stages of societal development intergroup collaboration is quickly destroyed, later in the more enlightened times it is not as readily recovered. During the initial 10^4^ periods, the subordinate group’s willingness to collaborate declines on average ~1.67·10^3^ times faster than it accrues beyond the point of enlightenment.

Indeed, Fig 7 shows that even if all members of the dominant group are repeatedly replaced with enlightened individuals, who do not discriminate between groups, i.e. ∀*i*,*t ρ*_*i*:*J*,*t*_ *= ρ*_*i*:*I*,*t*_, members of the subordinate group will continue to distrust members of the dominant group and exhibit an unwillingness to collaborate with them. The mechanism behind this is the same as that underlying persistence of stigma: over time the learning rate declines (see Fig 3), which causes the members of subordinate group to preserve their beliefs despite the increased collaboration from the members of the dominant group. Consequently, intergroup mistrust and non-collaboration persists. Note that Fig 7 shows an extreme case of instantaneous generational change one would never observe in a real society. If we assume a more realistic process of generational replacement, where members of the prejudiced dominant group are gradually replaced with enlightened individuals, the negative behavioral effects of initial stigmatization are even more resistant to change (see S3 Fig).

**Fig 8:**
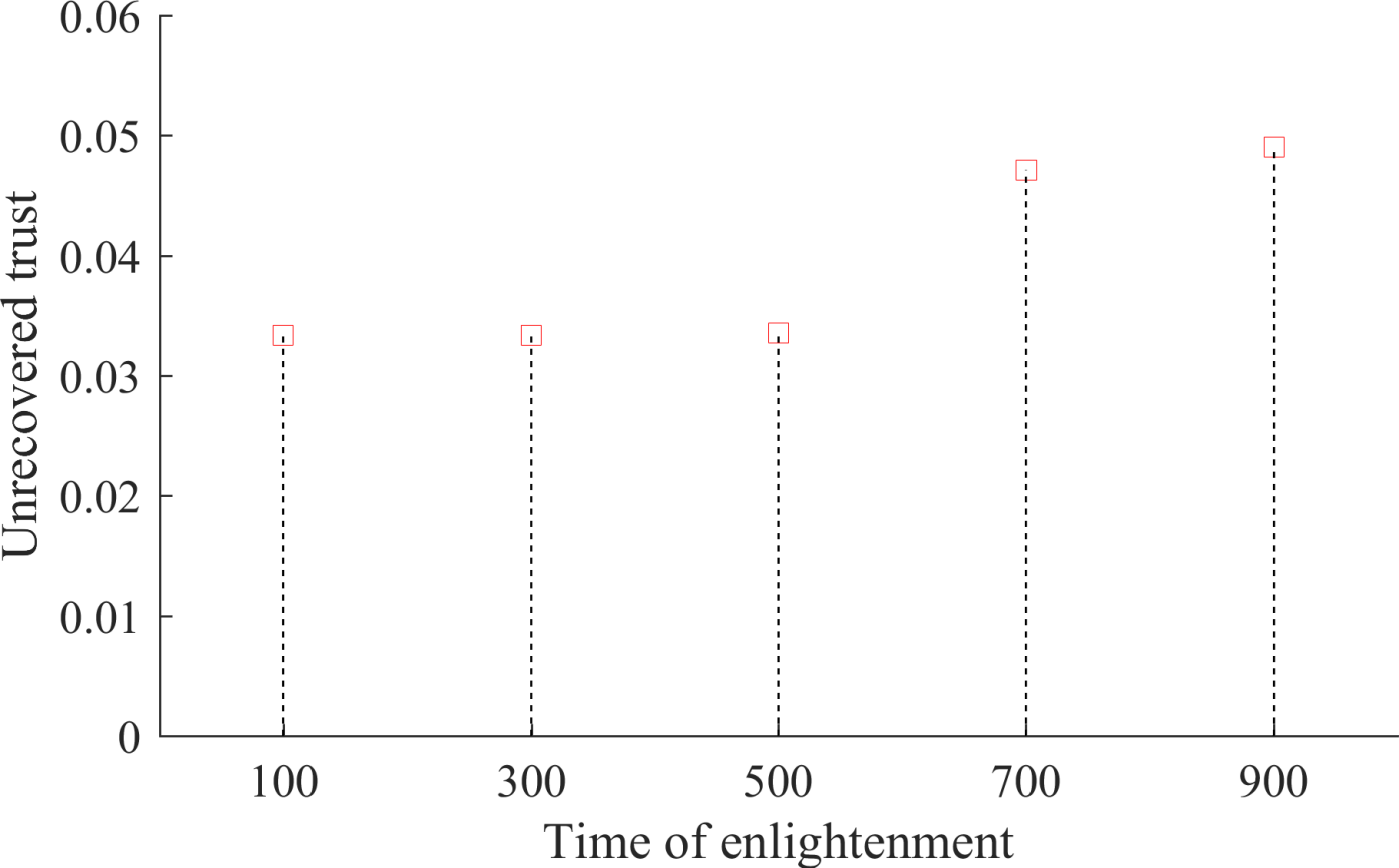
Effects of delayed enlightenment. The horizontal axis shows the time beyond which the prejudiced members of the dominant group are replaced with perfectly enlightened individuals. The vertical axis depicts the level of unrecovered trust, measured as the difference between dominant and subordinate group members’ willingness to collaborate at the period *t* = time of enlightenment + 10^7^.

Despite the difficulty of alleviating the negative effects of initial prejudices, however, we find that delaying efforts to do so entails further costs for society. Fig 8 shows that if society delays enlightenment, the amount of unrecoverable trust on the part of mistreated subordinate group members becomes even greater. Another neglectful generation, therefore, may cost the society additional losses in terms of intergroup collaboration.

## Simulating societies

Although agent-based models are of course artificial in nature, they have the benefit of examining how well-documented experimental effects play out over time, in populations of varying composition and with relevant parameters systematically manipulated in a manner that would be impossible in a real world experimental setting. The simple assumptions in our simulations are supported by copious experimental and field evidence [1, 12, 15]. Thus, albeit artificial, the models can inform our understanding of how known effects evolve over time in interaction with one another. The basic pattern of our results holds across a wide range of assumptions—we manipulate variables such as absolute and relative group size, high vs. low trust environment, distribution of initial beliefs, strength of initial beliefs, and speed of generational replacement, and show the key findings to be robust to these parameters (see S1 and S2 Files, S1–S12 Figs).

As in related prior work [11, 26], the comparisons to contemporary real societies are conceptual rather than literal, reproducing broad social patterns seen around the world rather than corresponding to empirical data on trust levels or income gaps in a single specific society. This follows on earlier work by [34], who shows that a few simple principles can reproduce the generally observed pattern of residential segregation across societies, without arguing this corresponds precisely to residential patterns in the United States, Malaysia, Russia, or Belgium.

A strength of the agent-based modeling is to show that a given set of select factors can be sufficient (albeit not necessary) to produce an outcome of interest. A core principle is therefore to include as few focal variables in the model as possible [35]. There are, however, numerous other psychological biases that could be considered in future research when modeling intergroup dynamics, such as generalization of prejudices from one social outgroup to another [8], the rationalization of prejudice via dissonance or self-perception [36], and discrepancies between perceived and actual norms [37, 38], among many others.

## Conclusion

Two subjective psychological factors, specifically 1) initial prejudice against a subordinate group among early members of a society’s dominant group and 2) the presence of some degree of conformity to the opinion of peers were sufficient to produce a sustained cycle of intergroup mistrust and non-collaboration in artificial societies. Even the subsequent replacement of all prejudiced dominant group members with non-prejudiced individuals was not enough to repair intergroup relations, with each additional generation of stigmatization leading to unrecoverable losses of intergroup collaboration. In real societies, the accumulation of objective gaps between groups in hereditary wealth, educational attainment, and human capital likely render the vicious cycle of intergroup discrimination and alienation even more intractable. These findings hold important implications for understanding the persistence of group-based inequality in so many human societies.

## Supporting information

S1 File Simulation code. Simulations were programmed in C++ using C++11 ISO standard.
(PDF)

S2 File Notes. S2 File details parameter values used in the supporting information.
(PDF)

S1 Fig Time until convergence as a function of learning speed *α*. S1 Fig shows how learning speed influences time until convergence. Point of convergence is measured as the earliest moment when the difference between dominant and subordinate group members’ willingness to trust reaches a certain threshold (without loss of generality set equal to 10^−4^). The graph demonstrates that faster speed of learning leads to more rapid convergence in behavior. As *α* decreases, the time to convergences increases exponentially. In the limit, when *α* → 0, agents do not learn and hence there is no convergence.
(TIF)

S2 Fig Comparative dynamics of *α* in small and large groups. S2 Fig shows the impact of group size on learning rates over time. In this experiment we assume no bias, i.e. *ρ*_*j*:*I*,*0*_ ~ *U*(0,1) and *ρ*_*i*:*J*,*0*_ ~ *U*(0,1). The difference in dynamics, therefore, is driven solely by the variance in the group size. On average, *α* of the subordinate group is always lower than that of the dominant group. In other words, members of the subordinate group come to share similar beliefs faster than members of the dominant group. A more numerous dominant group is less flexible in terms of its beliefs for two reasons: i) an individual’s learning has a smaller effect on the group’s opinion and ii) majority group members are less likely to interact with minorities than vice versa.
(TIF)

S3 Fig Gradual generational replacement. S3 Fig shows the effect of gradually replacing dominant group members with enlightened individuals. We assume that 1 randomly chosen member is replaced every 100 periods. This process serves as a rough approximation for birth and death in an actual human society. It is further aligned with the empirical observation [39] that major shifts in societal values occur primarily through generational replacement (e.g., older, prejudiced individuals dying and being replaced by younger, less prejudiced individuals). Generational replacement fails to eliminate the distrust members of the subordinate group have in the dominant group.
(TIF)

S4 Fig Inverted group sizes. S4 Fig shows the dynamics of the willingness to cooperate in a society where the stigmatized subordinate group is numerically larger in size than the dominant group. Specifically, we assume that |*I*| = 20 and |*J*| = 80. Inverting the relative size of the groups has no qualitative effects on the persistence of stigma.
(TIF)

S5 Fig Collaborative population. S5 Fig shows the dynamics of willingness to trust in a population where individuals initially generally tend to collaborate in social interactions. Specifically, we set *ρ*_*j*:*I*,*0*_ ~ *U*(0.5,1) and *ρ*_*i*:*J*,*0*_ ~ *U*(0.5,0.75). In a collaborative population, like in the neutral ones examined in the main text, social conformity prevents the disappearance of stigma.
(TIF)

S6 Fig Distrustful population. S6 Fig shows the dynamics of intergroup collaboration in a population where members initially tend to distrust each other. Specifically, we set *ρ*_*j*:*I*,*0*_ ~ *U*(0,0.5) and *ρ*_*i*:*J*,*0*_ ~ *U*(0,0.25). S6 Fig shows that low levels of trust in the population do not change the main qualitative observations.
(TIF)

S7 Fig Truncated normal distribution. S7 Fig show the dynamics of willingness to trust when initial beliefs follow a truncated normal, and not uniform, distribution. Specifically, Panels A through D depict the dynamics of intergroup collaboration for the following initial distributions: A) *ρ*_*j*:*I*,*0*_ ~ *N*(0.5,0.1) | *ρ*_*j*:*I*,*0*_ ∈ (0,1) and *ρ*_*i*:*J*,*0*_ ~ *N*(0.25,0.1) | *ρ*_*j*:*I*,*0*_ ∈ (0,1), B) *ρ*_*j*:*I*,*0*_ ~ *N*(0.5,0.2) | *ρ*_*j*:*I*,*0*_ ∈ (0,1) and *ρ*_*i*:*J*,*0*_ ~ *N*(0.25,0.2) | *ρ*_*j*:*I*,*0*_ ∈ (0,1), C) *ρ*_*j*:*I*,*0*_ ~ *N*(0.5,0.3) | *ρ*_*j*:*I*,*0*_ ∈ (0,1) and *ρ*_*i*:*J*,*0*_ ~ *N*(0.25,0.3) | *ρ*_*j*:*I*,*0*_ ∈ (0,1), and D) *ρ*_*j*:*I*,*0*_ ~ *N*(0.5,0.4) | *ρ*_*j*:*I*,*0*_ ∈ (0,1) and *ρ*_*i*:*J*,*0*_ ~ *N*(0.25,0.4) | *ρ*_*j*:*I*,*0*_ ∈ (0,1). S7 Fig show that when the variance is large (D) the initial stigma is relatively small as the initial willingness to collaborate among dominant group members is comparatively high. This is an outcome of asymmetric truncation (mean = 0.25, distance to lower truncation = 0.25, distance to the upper truncation = 0.75). As the variance declines (D → A), stigma against the subordinate group becomes greater. Regardless of the level of variance, results of simulations with a truncated normal distribution of initial beliefs are consistent with our main observations (see Fig 4).
(TIF)

S8 Fig Large population with generational replacement. S8 Fig shows the consequences of perfect enlightenment at some distant moment in time. Specifically, we set enlightenment at *t* ≥ 10^5^ (note that to preserve comparability with Fig 5 we keep the average count of accumulated interactions per individual constant at 10^2^); beyond this moment all dominant group members do not see any difference between dominant and subordinate group members, i.e. do not discriminate. The main observation is that even perfect enlightenment, when put into action at such a late stage in societal development, cannot revive intergroup trust (cf. Fig 5).
(TIF)

S9 Fig Large population of predominantly collaborative members. S9 Fig shows the dynamics of willingness to trust in a generally collaborative population. In this experiment we constrain individuals to collaborate with one another in social interactions. Specifically, we set *ρ*_*j*:*I*,*0*_ ~ *U*(0.5,1) and *ρ*_*i*:*J*,*0*_ ~ *U*(0.5,0.75). High levels of trust in a large population do not change the main qualitative observations.
(TIF)

S10 Fig Large population of predominantly distrustful individuals. S10 Fig shows the dynamics of intergroup collaboration in a population where individuals initially tend to distrust each other. Specifically, we set *ρ*_*j*:*I*,*0*_ ~ *U*(0,0.5) and *ρ*_*i*:*J*,*0*_ ~ *U*(0,0.25). Low levels of trust in a large population do not change the main qualitative observations.
(TIF)

S11 Fig Large population, with group sizes inverted. S11 Fig shows the dynamics of the willingness to cooperate in a society where the stigmatized subordinate group is actually numerically larger in size than the dominant group. Specifically, we assume that |*I*| = 200 and |*J*| = 800. Inversing the relative size of the groups in a large population has no qualitative effects on the persistence of stigma.
(TIF)

S12 Fig Large population with truncated normal distribution of initial beliefs. S12 Fig shows the dynamics of collaboration in a population where initial beliefs are distributed normally. Specifically, we set *ρ*_*j*:*I*,*0*_ ~ *N*(0.5,0.2) | *ρ*_*j*:*I*,*0*_ ∈ (0,1) and *ρ*_*i*:*J*,*0*_ ~ *N*(0.25,0.2) | *ρ*_*j*:*I*,*0*_ ∈ (0,1). As seen in S12 Fig, the main findings continue to hold.
(TIF)
