# Supplementary material for: Initial prejudices create cross-generational intergroup mistrust
Source: PLoS One. 2018 Apr 25;13(4):e0194871. doi: 10.1371/journal.pone.0194871 (PMC5918755; doi:10.1371/journal.pone.0194871)
Supplement: S1 File — Simulations were programmed in C++ using C++11 ISO standard. (PDF) [file pone.0194871.s001.pdf]

The data that were generated with this code are posted on the Open Science Framework at: https://osf.io/452nr/?view_only=75325ed7615841a69a6d4b04c0aa8058. The Figures in the main body of the paper and this supplement were produced using these data.

```
#include <iostream>
#include <cstdlib>
#include <ctime>
#include <cmath>
#include <fstream>
#include <algorithm>
#include <cfloat>
#include <random>
#include <chrono>

using namespace std;

// Random seeding
unsigned int seed = (unsigned int) chrono::system_clock::now().time_since_epoch().count();
mt19937 generator(seed);

// Global constants
const int       N_RUNS = 100;               // Number of general simulation runs (result will be averaged)
const int       N_EPOCHS = 100000;           // Number of evaluation epochs
const int       MAJORITY_SIZE = 80;         // Size of the majority set
const int       MINORITY_SIZE = 20;         // Size of the minority set
const int       N_PLAYS_PER_EPOCH = 100;    // Number of simulation steps per epoch
const int       GENERATION_CHANGE_POINT = 1000000; // The moment of generational replacement in the base case is beyond the observed time period.

// Parameters of the distribution for agents initialization
const double    unbiased = 1;
const double    bias = 0.5;
const double    cooperativeness = 0;

enum AGENT_TYPE
{
   MAJORITY = 0,
   MINORITY = 1
};

double Majority[2][MAJORITY_SIZE];
double Minority[2][MINORITY_SIZE];
double Dynamics[5][N_EPOCHS];
double Average_alpha[2];

// Indices of current agents
int first, second;
// agent_type means the type of the first player, i.e. MAJORITY if majority and MINORITY if minority.
```

```
int agent_type[2];

//Declaration of the functions (note: depending on the specification different functions may
be idle). All of these functions are sequentially defined below
void Initiate_Willingness_to_Cooperate();
void Learn(int strategy_1, int strategy_2);
void Play();
void Select_Players();
double Measure_Population_Willingness_to_Cooperate();
void Initiate_Dynamics();
double Measure_Majority_0_WTC();
double Measure_Majority_1_WTC();
double Measure_Minority_0_WTC();
double Measure_Minority_1_WTC();
double Measure_Majority_0_common();
double Measure_Majority_1_common();
double Measure_Minority_0_common();
double Measure_Minority_1_common();
void Generation_Change();
void Measure_Average_alpha();

int main()//Start of simulation
{
   ofstream myfile;
   Initiate_Dynamics(); //Assuring that nothing is stored in the memory

   for(int run = 0; run < N_RUNS; run++) //Initiating the loop for the number of simulations
   {
      cout << "Running simulation step " << run+1 << " of " << N_RUNS << "... " << endl;
      int prevCompletionPercent = -1;

      Initiate_Willingness_to_Cooperate(); //Creating initial sets of agents
      for (int tt = 0; tt < N_EPOCHS; tt++) // Time loop 1
      {
         Dynamics[0][tt] = Dynamics[0][tt] +
Measure_Population_Willingness_to_Cooperate();
         Dynamics[1][tt] = Dynamics[1][tt] + Measure_Majority_0_WTC();
         Dynamics[2][tt] = Dynamics[2][tt] + Measure_Majority_1_WTC();
         Dynamics[3][tt] = Dynamics[3][tt] + Measure_Minority_0_WTC();
         Dynamics[4][tt] = Dynamics[4][tt] + Measure_Minority_1_WTC();
         //Measure_Average_alpha();
         //Dynamics[2][tt] = Dynamics[2][tt] + Average_alpha[0];
         //Dynamics[3][tt] = Dynamics[3][tt] + Average_alpha[1];
         for (int t = 0; t < N_PLAYS_PER_EPOCH; t++)// Time loop 2
         {
            if (tt >= GENERATION_CHANGE_POINT) //Execute generational replacement
when applicable
            {
               Generation_Change();
            }
```

```
100                 Select_Players();
101                 Play();
102             }
103             // Print completion percent
104             int completionPercent = int(double(tt) / (N_EPOCHS - 1) * 100);
105             if (completionPercent != prevCompletionPercent && completionPercent % 10 == 0)
106     {
107                 cout << completionPercent << "%  ";
108                 prevCompletionPercent = completionPercent;
109             }
110         }
111
112         cout << endl << endl;
113     }
114     //Storing the data. For the ease of processing, we store only average values.
115     remove("0.txt");
116     myfile.open("0.txt", ofstream::app);
117     for (int tt = 0; tt < N_EPOCHS; tt++)
118     {
119         myfile << Dynamics[0][tt] / N_RUNS << ";";
120     }
121     myfile.close();
122     remove("1.txt");
123     myfile.open("1.txt", ofstream::app);
124     for (int tt = 0; tt < N_EPOCHS; tt++)
125     {
126         myfile << Dynamics[1][tt] / N_RUNS << ";";
127     }
128     myfile.close();
129     remove("2.txt");
130     myfile.open("2.txt", ofstream::app);
131     for (int tt = 0; tt < N_EPOCHS; tt++)
132     {
133         myfile << Dynamics[2][tt] / N_RUNS << ";";
134     }
135     myfile.close();
136     remove("3.txt");
137     myfile.open("3.txt", ofstream::app);
138     for (int tt = 0; tt < N_EPOCHS; tt++)
139     {
140         myfile << Dynamics[3][tt] / N_RUNS << ";";
141     }
142     myfile.close();
143     remove("4.txt");
144     myfile.open("4.txt", ofstream::app);
145     for (int tt = 0; tt < N_EPOCHS; tt++)
146     {
147         myfile << Dynamics[4][tt] / N_RUNS << ";";
148     }
149     myfile.close();
```

```
150     cout << '\a';
151
152     return 0;
153   }//End of simulation
154
155   void Initiate_Willingness_to_Cooperate()//Define the initial population
156   {
157     //normal_distribution<double> distribution(unbiased, cooperativeness);
158     //normal_distribution<double> biased_distribution(bias*unbiased, cooperativeness);
159     uniform_real_distribution<double> distribution(cooperativeness, unbiased);
160     uniform_real_distribution<double> biased_distribution(cooperativeness, bias*unbiased);
161
162     for (int i = 0; i < MAJORITY_SIZE; i++)
163     {
164       Majority[0][i] = distribution(generator);
165       if (Majority[0][i] > 1 || Majority[0][i] < 0)
166       {
167         i--;
168       }
169     }
170     for (int i = 0; i < MAJORITY_SIZE; i++)
171     {
172       Majority[1][i] = biased_distribution(generator);
173       if (Majority[1][i] > 1 || Majority[1][i] < 0)
174       {
175         i--;
176       }
177     }
178     for (int i = 0; i < MINORITY_SIZE; i++)
179     {
180       Minority[0][i] = distribution(generator);
181       Minority[1][i] = distribution(generator);
182       if (Minority[0][i] > 1 || Minority[0][i] < 0 || Minority[1][i] > 1 || Minority[1][i] < 0)
183       {
184         i--;
185       }
186     }
187   }
188
189   // Select two random players from the population
190   void Select_Players()
191   {
192     uniform_real_distribution<double> uniform(0, 1);
193
194     if (uniform(generator) < (double)MAJORITY_SIZE / (MAJORITY_SIZE +
195   MINORITY_SIZE))
196     {
197       agent_type[0] = MAJORITY;
198     }
199     else
```

```
200     {
201         agent_type[0] = MINORITY;
202     }
203     if (uniform(generator) < (double)MAJORITY_SIZE / (MAJORITY_SIZE +
204 MINORITY_SIZE))
205     {
206         agent_type[1] = MAJORITY;
207     }
208     else
209     {
210         agent_type[1] = MINORITY;
211     }
212
213     // Make two uniform distrubutions (one for each agent),
214     // Each has a maximum value as the size of the proper set of agents
215     uniform_int_distribution<int> select_first(0, (1 - agent_type[0]) * MAJORITY_SIZE +
216 agent_type[0] * MINORITY_SIZE - 1);
217     uniform_int_distribution<int> select_second(0, (1 - agent_type[1]) * MAJORITY_SIZE +
218 agent_type[1] * MINORITY_SIZE - 1);
219
220     // Select first agent
221     first = select_first(generator);
222
223     // Select second agent.
224     // If types of agents are the same then make sure that agentes are different
225     if (agent_type[0] == agent_type[1])
226     {
227         do
228         {
229             second = select_second(generator);
230         } while (second == first);
231     }
232     else
233     {
234         second = select_second(generator);
235     }
236 }
237
238 // Simulate formation of belief and the choice of strategies by both players
239 void Play()
240 {
241     uniform_real_distribution<double> uniform(0, 1);
242     int strategy_1 = 1;
243     int strategy_2 = 1;
244
245     if (agent_type[0] == MAJORITY)
246     {
247         if (agent_type[1] == MAJORITY)
248         {
249             if (uniform(generator) > Majority[0][first])
```

```
250             {
251                 strategy_1 = 0;
252             }
253             if (uniform(generator) > Majority[0][second])
254             {
255                 strategy_2 = 0;
256             }
257         }
258         else
259         {
260             if (uniform(generator) > Majority[1][first])
261             {
262                 strategy_1 = 0;
263             }
264             if (uniform(generator) > Minority[0][second])
265             {
266                 strategy_2 = 0;
267             }
268         }
269     }
270     else
271     {
272         if (agent_type[1] == MAJORITY)
273         {
274             if (uniform(generator) > Minority[0][first])
275             {
276                 strategy_1 = 0;
277             }
278             if (uniform(generator) > Majority[1][second])
279             {
280                 strategy_2 = 0;
281             }
282         }
283         else
284         {
285             if (uniform(generator) > Minority[1][first])
286             {
287                 strategy_1 = 0;
288             }
289             if (uniform(generator) > Minority[1][second])
290             {
291                 strategy_2 = 0;
292             }
293         }
294     }
295
296     Learn(strategy_1, strategy_2);
297 }
298
299 // Adjustment of belief upon interaction
```

```
300     void Learn(int strategy_1, int strategy_2)
301     {
302        double alpha_1;
303        double alpha_2;
304
305        uniform_real_distribution<double> distribution(0, 1);
306        if (agent_type[0] == MAJORITY)
307        {
308           if (agent_type[1] == MAJORITY)
309           {
310              double common = Measure_Majority_0_common();
311              alpha_1 = abs(common - Majority[0][first]);
312              alpha_2 = abs(common - Majority[0][second]);
313              Majority[0][first] = alpha_1*strategy_2 + (1 - alpha_1)*Majority[0][first];
314              Majority[0][second] = alpha_2*strategy_1 + (1 - alpha_2)*Majority[0][second];
315           }
316           else
317           {
318              alpha_1 = abs(Measure_Majority_1_common() - Majority[1][first]);
319              alpha_2 = abs(Measure_Minority_0_common() - Minority[0][second]);
320              Majority[1][first] = alpha_1*strategy_2 + (1 - alpha_1)*Majority[1][first];
321              Minority[0][second] = alpha_2*strategy_1 + (1 - alpha_2)*Minority[0][second];
322           }
323        }
324        else
325        {
326           if (agent_type[1] == MAJORITY)
327           {
328              alpha_1 = abs(Measure_Minority_0_common() - Minority[0][first]);
329              alpha_2 = abs(Measure_Majority_1_common() - Majority[1][second]);
330              Minority[0][first] = alpha_1*strategy_2 + (1 - alpha_1)*Minority[0][first];
331              Majority[1][second] = alpha_2*strategy_1 + (1 - alpha_2)*Majority[1][second];
332           }
333           else
334           {
335              double common = Measure_Minority_1_common();
336              alpha_1 = abs(common - Minority[1][first]);
337              alpha_2 = abs(common - Minority[1][second]);
338              Minority[1][first] = alpha_1*strategy_2 + (1 - alpha_1)*Minority[1][first];
339              Minority[1][second] = alpha_2*strategy_1 + (1 - alpha_2)*Minority[1][second];
340           }
341        }
342     }
343     // Measure average willingness to cooperate in the entire population
344     double Measure_Population_Willingness_to_Cooperate()
345     {
346        double average_1 = 0;
347        double average_2 = 0;
348        for (int i = 0; i < MAJORITY_SIZE; i++)
349        {
```

```
        average_1 = average_1 + Majority[0][i] * MAJORITY_SIZE / (MAJORITY_SIZE +
MINORITY_SIZE);
        average_1 = average_1 + Majority[1][i] * MINORITY_SIZE / (MAJORITY_SIZE +
MINORITY_SIZE);
    }
    for (int i = 0; i < MINORITY_SIZE; i++)
    {
        average_2 = average_2 + Minority[0][i] * MAJORITY_SIZE / (MAJORITY_SIZE +
MINORITY_SIZE);
        average_2 = average_2 + Minority[1][i] * MINORITY_SIZE / (MAJORITY_SIZE +
MINORITY_SIZE);
    }
    average_1 = average_1 / MAJORITY_SIZE;
    average_2 = average_2 / MINORITY_SIZE;

    return average_1*MAJORITY_SIZE / (MAJORITY_SIZE + MINORITY_SIZE) +
average_2*MINORITY_SIZE / (MAJORITY_SIZE + MINORITY_SIZE);
}

// Measure majority's average willingness to cooperate with majority
double Measure_Majority_0_WTC()
{
    double average = 0;
    for (int i = 0; i < MAJORITY_SIZE; i++)
    {
        average = average + Majority[0][i];
    }

    return average / MAJORITY_SIZE;
}

// Measure majority's social belief about their own willingness to cooperate
double Measure_Majority_0_common()
{
    double sum = 0;

    double Deviation[MAJORITY_SIZE];

    for (int i = 0; i < MAJORITY_SIZE; i++)
    {
        Deviation[i] = 0;
        for (int j = 0; j < MAJORITY_SIZE; j++)
        {
            if (i != j)
            {
                Deviation[i] = Deviation[i] + abs(Majority[0][i] - Majority[0][j]) +
DBL_EPSILON;
            }
        }
        sum = sum + 1.0 / Deviation[i];
```

```
400         }
401         double common_belief = 0;
402         for (int i = 0; i < MAJORITY_SIZE; i++)
403         {
404             common_belief = common_belief + Majority[0][i] * (1.0 / Deviation[i]) / sum;
405         }
406
407         return common_belief;
408     }
409
410     // Measure majority's average willingness to cooperate with minority
411     double Measure_Majority_1_WTC()
412     {
413         double average = 0;
414         for (int i = 0; i < MAJORITY_SIZE; i++)
415         {
416             average = average + Majority[1][i];
417         }
418
419         return average / MAJORITY_SIZE;
420     }
421
422     // Measure majority's belief about minority's willingness to cooperate
423     double Measure_Majority_1_common()
424     {
425         double sum = 0;
426
427         double Deviation[MAJORITY_SIZE];
428
429         for (int i = 0; i < MAJORITY_SIZE; i++)
430         {
431             Deviation[i] = 0;
432             for (int j = 0; j < MAJORITY_SIZE; j++)
433             {
434                 if (i != j)
435                 {
436                     Deviation[i] = Deviation[i] + abs(Majority[1][i] - Majority[1][j]) +
437     DBL_EPSILON;
438                 }
439             }
440             sum = sum + 1.0 / Deviation[i];
441         }
442         double common_belief = 0;
443         for (int i = 0; i < MAJORITY_SIZE; i++)
444         {
445             common_belief = common_belief + Majority[1][i] * (1.0 / Deviation[i]) / sum;
446         }
447
448         return common_belief;
449     }
```

```
450
451     // Measure minority's average willingness to cooperate with minority
452     double Measure_Minority_0_WTC()
453     {
454        double average = 0;
455        for (int i = 0; i < MINORITY_SIZE; i++)
456        {
457           average = average + Minority[0][i];
458        }
459
460        return average / MINORITY_SIZE;
461     }
462
463     // Measure minority's belief about majority's willingness to cooperate
464     double Measure_Minority_0_common()
465     {
466        double sum = 0;
467        double Deviation[MINORITY_SIZE];
468
469        for (int i = 0; i < MINORITY_SIZE; i++)
470        {
471           Deviation[i] = 0;
472           for (int j = 0; j < MINORITY_SIZE; j++)
473           {
474              if (i != j)
475              {
476                 Deviation[i] = Deviation[i] + abs(Minority[0][i] - Minority[0][j]) +
477     DBL_EPSILON;
478              }
479           }
480           sum = sum + 1.0 / Deviation[i];
481        }
482        double common_belief = 0;
483        for (int i = 0; i < MINORITY_SIZE; i++)
484        {
485           common_belief = common_belief + Minority[0][i] * (1.0 / Deviation[i]) / sum;
486        }
487
488        return common_belief;
489     }
490
491     // Measure minority's average willingness to cooperate with minority
492     double Measure_Minority_1_WTC()
493     {
494        double average = 0;
495        for (int i = 0; i < MINORITY_SIZE; i++)
496        {
497           average = average + Minority[1][i];
498        }
499
```

```
500     return average / MINORITY_SIZE;
501 }
502
503 // Measure minority's belief about their own willingness to cooperate
504 double Measure_Minority_1_common()
505 {
506     double sum = 0;
507
508     double Deviation[MINORITY_SIZE];
509
510     for (int i = 0; i < MINORITY_SIZE; i++)
511     {
512         Deviation[i] = 0;
513         for (int j = 0; j < MINORITY_SIZE; j++)
514         {
515             if (i != j)
516             {
517                 Deviation[i] = Deviation[i] + abs(Minority[1][i] - Minority[1][j]) +
518 DBL_EPSILON;
519             }
520         }
521         sum = sum + 1.0 / Deviation[i];
522     }
523     double common_belief = 0;
524     for (int i = 0; i < MINORITY_SIZE; i++)
525     {
526         common_belief = common_belief + Minority[1][i] * (1.0 / Deviation[i]) / sum;
527     }
528
529     return common_belief;
530 }
531
532 void Initiate_Dynamics()
533 {
534     for (int tt = 0; tt < N_EPOCHS; tt++)
535     {
536         Dynamics[0][tt] = 0;
537         Dynamics[1][tt] = 0;
538         Dynamics[2][tt] = 0;
539         Dynamics[3][tt] = 0;
540         Dynamics[4][tt] = 0;
541     }
542 }
543
544 void Generation_Change()
545 {
546     uniform_int_distribution<int> distribution(0, MAJORITY_SIZE - 1);
547     //
548     for (int i = 0; i < MAJORITY_SIZE; i++)
549     {
```

```
550         Majority[1][i] = Majority[0][i];
551      }
552      /*
553      for(int g = 0; g < 1; g++)
554      {
555         int i = distribution(generator);
556         Majority[1][i] = Majority[0][i];
557      }
558      */
559   }
560
561   // Measure average alpha for Majority[1] and Minority[0]
562   void Measure_Average_alpha()
563   {
564      Average_alpha[0] = 0;
565      Average_alpha[1] = 0;
566      double common = Measure_Majority_1_common();
567      for (int i = 0; i < MAJORITY_SIZE; i++)
568      {
569         Average_alpha[0] = Average_alpha[0] + abs(common - Majority[1][i]);
570      }
571      Average_alpha[0] = Average_alpha[0] / MAJORITY_SIZE;
572      common = Measure_Minority_0_common();
573      for (int i = 0; i < MINORITY_SIZE; i++)
574      {
575         Average_alpha[1] = Average_alpha[1] + abs(common - Minority[0][i]);
576      }
577      Average_alpha[1] = Average_alpha[1] / MINORITY_SIZE;
578   }
```
