# Supplementary material for: Initial prejudices create cross-generational intergroup mistrust
Source: PLoS One. 2018 Apr 25;13(4):e0194871. doi: 10.1371/journal.pone.0194871 (PMC5918755; doi:10.1371/journal.pone.0194871)
Supplement: S2 File — details parameter values used in the supporting information. (PDF) [file pone.0194871.s002.pdf]

For Figs S3 through S9, unless otherwise specified, we use the following parameter values: $|I| = 80$, $|J| = 20$, $T = 10^7$, $\rho_{j:I,0} \sim U(0,1)$ and $\rho_{i:J,0} \sim U(0,0.5)$, I stands for the dominant group and J denotes the subordinate group; presented results are averages from 100 independent runs in the presence of social conformity and 1000 runs otherwise. Figs S10 through S14 present further simulation results for a large population. Specifically we set |dominant| = 800, |subordinate| = 200. Presented results are averages from 10 independent runs.
