# Supplementary figures and images for: Initial prejudices create cross-generational intergroup mistrust
Source: PLoS One. 2018 Apr 25;13(4):e0194871. doi: 10.1371/journal.pone.0194871 (PMC5918755; doi:10.1371/journal.pone.0194871)

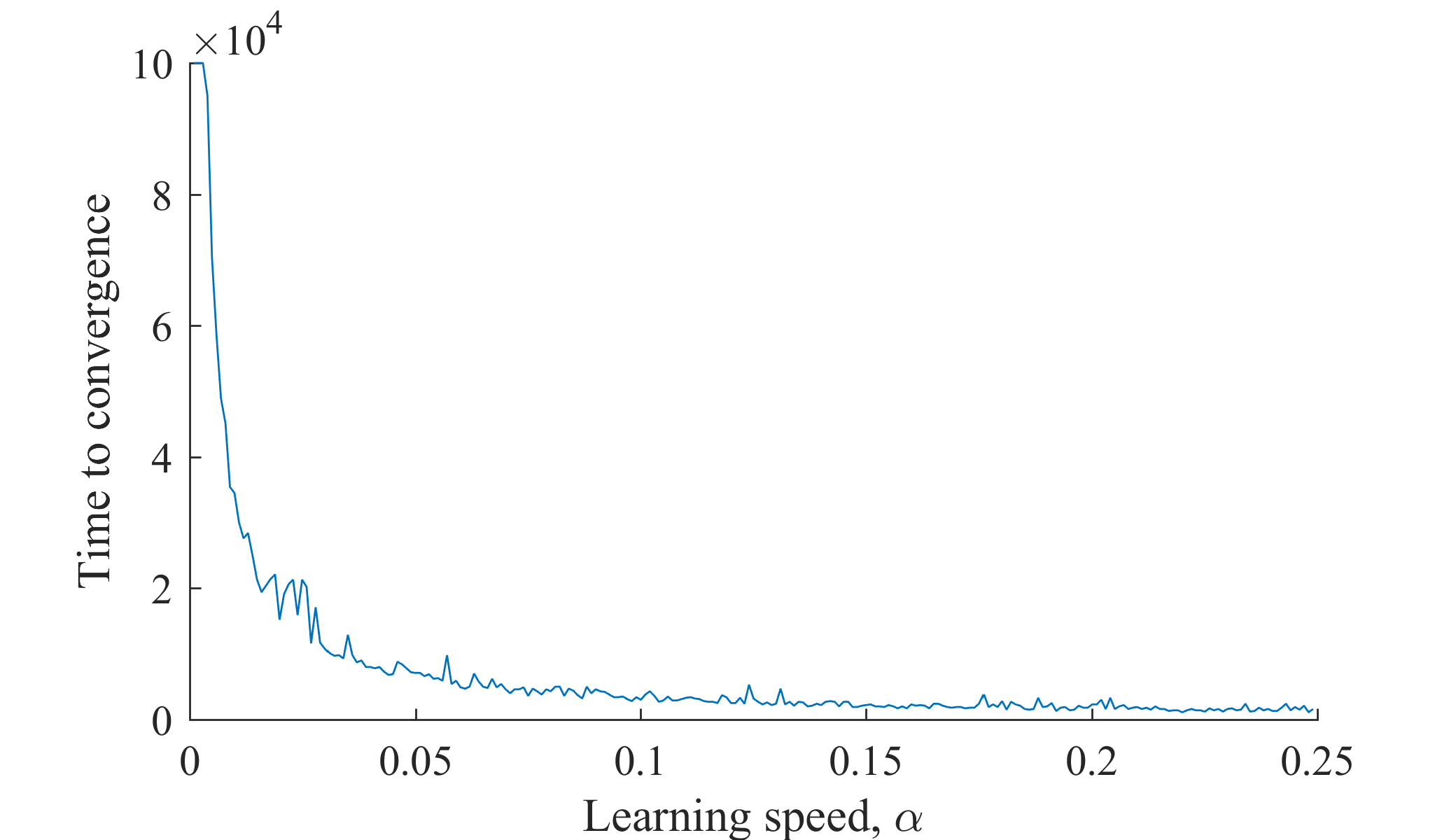

Supplement: S1 Fig — shows how learning speed influences time until convergence. Point of convergence is measured as the earliest moment when the difference between dominant and subordinate group members’ willingness to trust reaches a certain threshold (without loss of generality set equal to 10−4). The graph demonstrates that faster speed of learning leads to more rapid convergence in behavior. As α decreases, the time to convergences increases exponentially. In the limit, when α → 0, agents do not learn and hence there is no convergence. (TIF) [file pone.0194871.s003.tif]

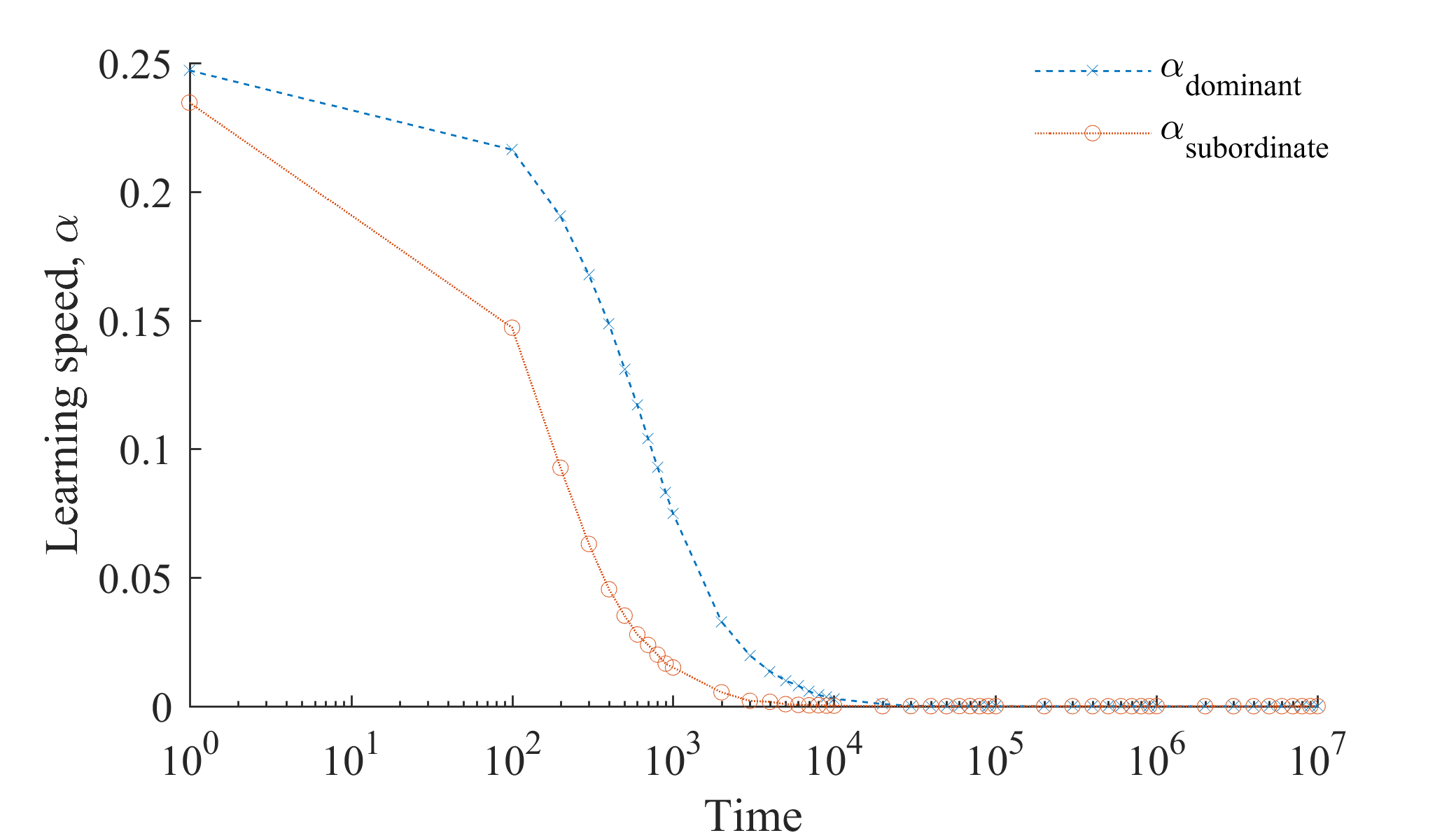

Supplement: S2 Fig — shows the impact of group size on learning rates over time. In this experiment we assume no bias, i.e. ρj:I,0 ~ U(0,1) and ρi:J,0 ~ U(0,1). The difference in dynamics, therefore, is driven solely by the variance in the group size. On average, α of the subordinate group is always lower than that of the dominant group. In other words, members of the subordinate group come to share similar beliefs faster than members of the dominant group. A more numerous dominant group is less flexible in terms of its beliefs for two reasons: i) an individual’s learning has a smaller effect on the group’s opinion and ii) majority group members are less likely to interact with minorities than vice versa. (TIF) [file pone.0194871.s004.tif]

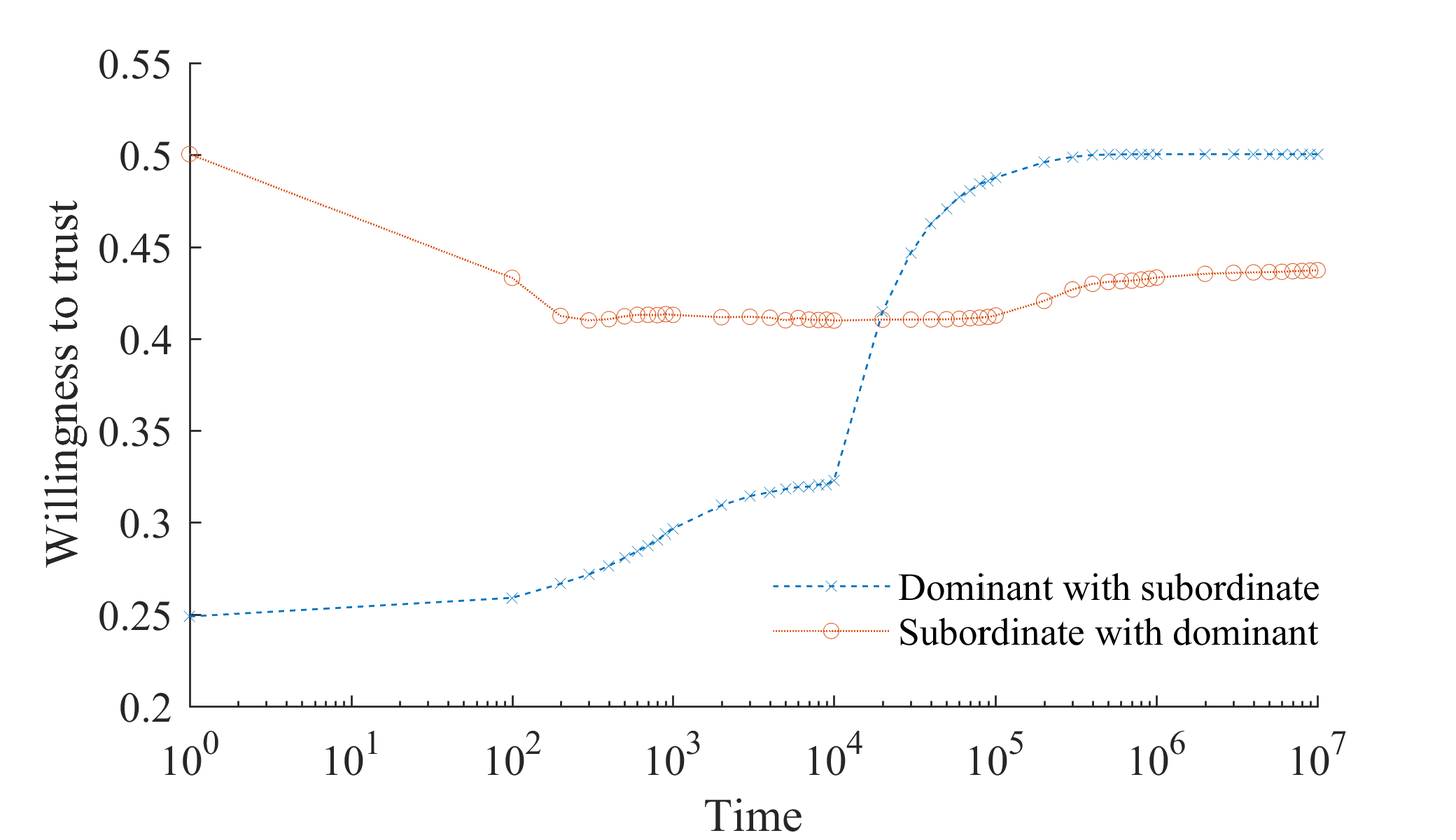

Supplement: S3 Fig — shows the effect of gradually replacing dominant group members with enlightened individuals. We assume that 1 randomly chosen member is replaced every 100 periods. This process serves as a rough approximation for birth and death in an actual human society. It is further aligned with the empirical observation [39] that major shifts in societal values occur primarily through generational replacement (e.g., older, prejudiced individuals dying and being replaced by younger, less prejudiced individuals). Generational replacement fails to eliminate the distrust members of the subordinate group have in the dominant group. (TIF) [file pone.0194871.s005.tif]

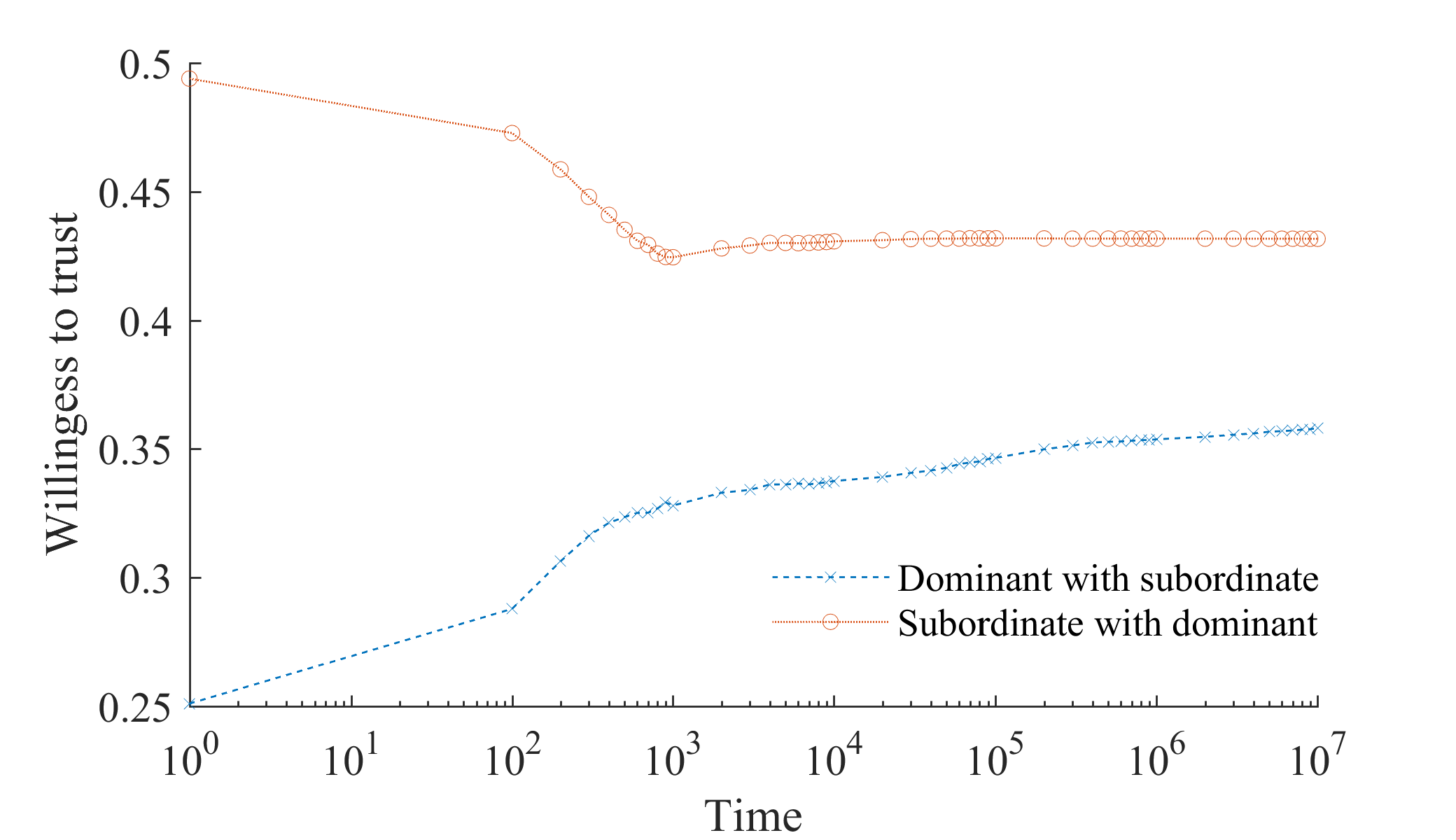

Supplement: S4 Fig — shows the dynamics of the willingness to cooperate in a society where the stigmatized subordinate group is numerically larger in size than the dominant group. Specifically, we assume that |I| = 20 and |J| = 80. Inverting the relative size of the groups has no qualitative effects on the persistence of stigma. (TIF) [file pone.0194871.s006.tif]

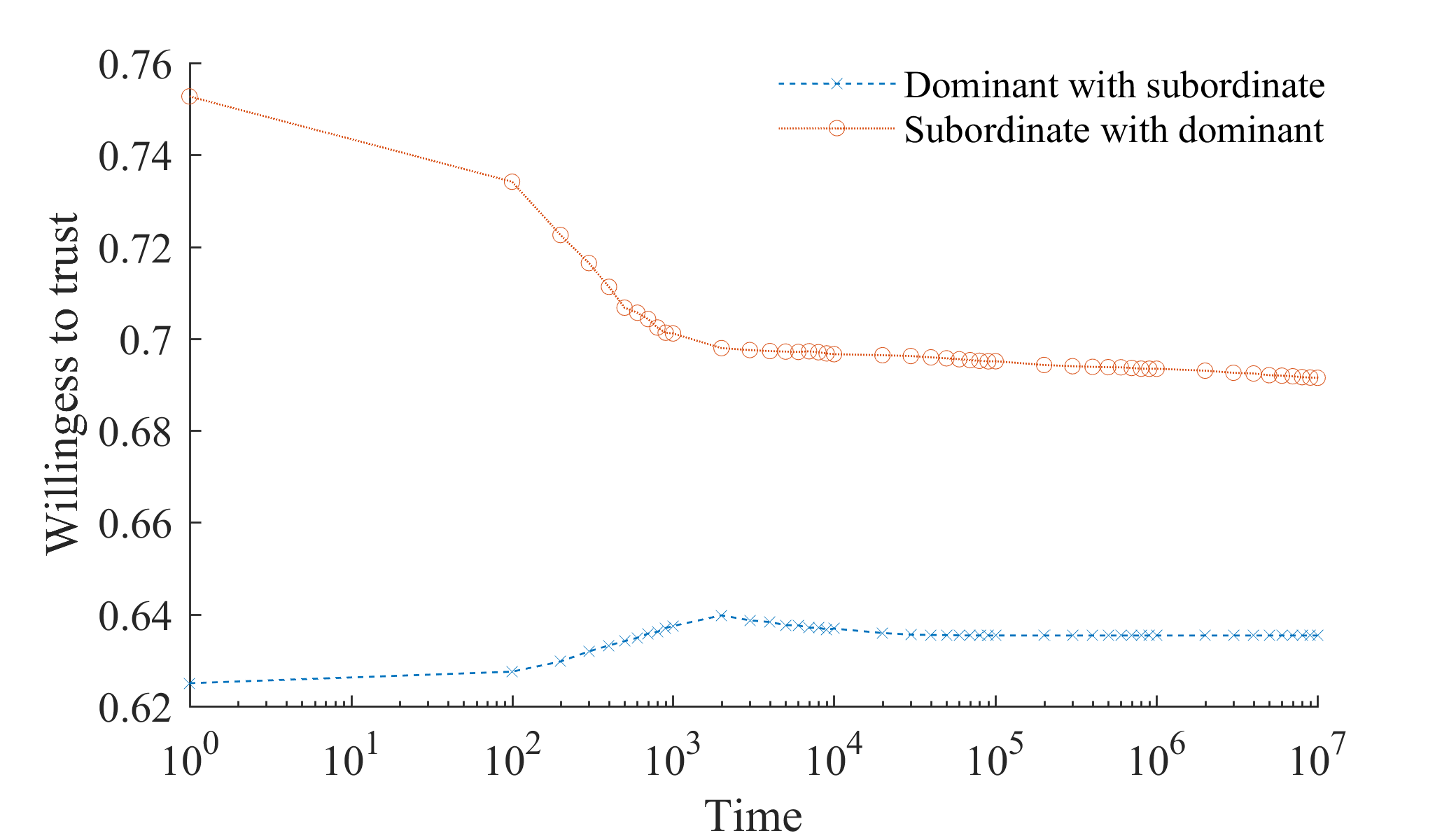

Supplement: S5 Fig — shows the dynamics of willingness to trust in a population where individuals initially generally tend to collaborate in social interactions. Specifically, we set ρj:I,0 ~ U(0.5,1) and ρi:J,0 ~ U(0.5,0.75). In a collaborative population, like in the neutral ones examined in the main text, social conformity prevents the disappearance of stigma. (TIF) [file pone.0194871.s007.tif]

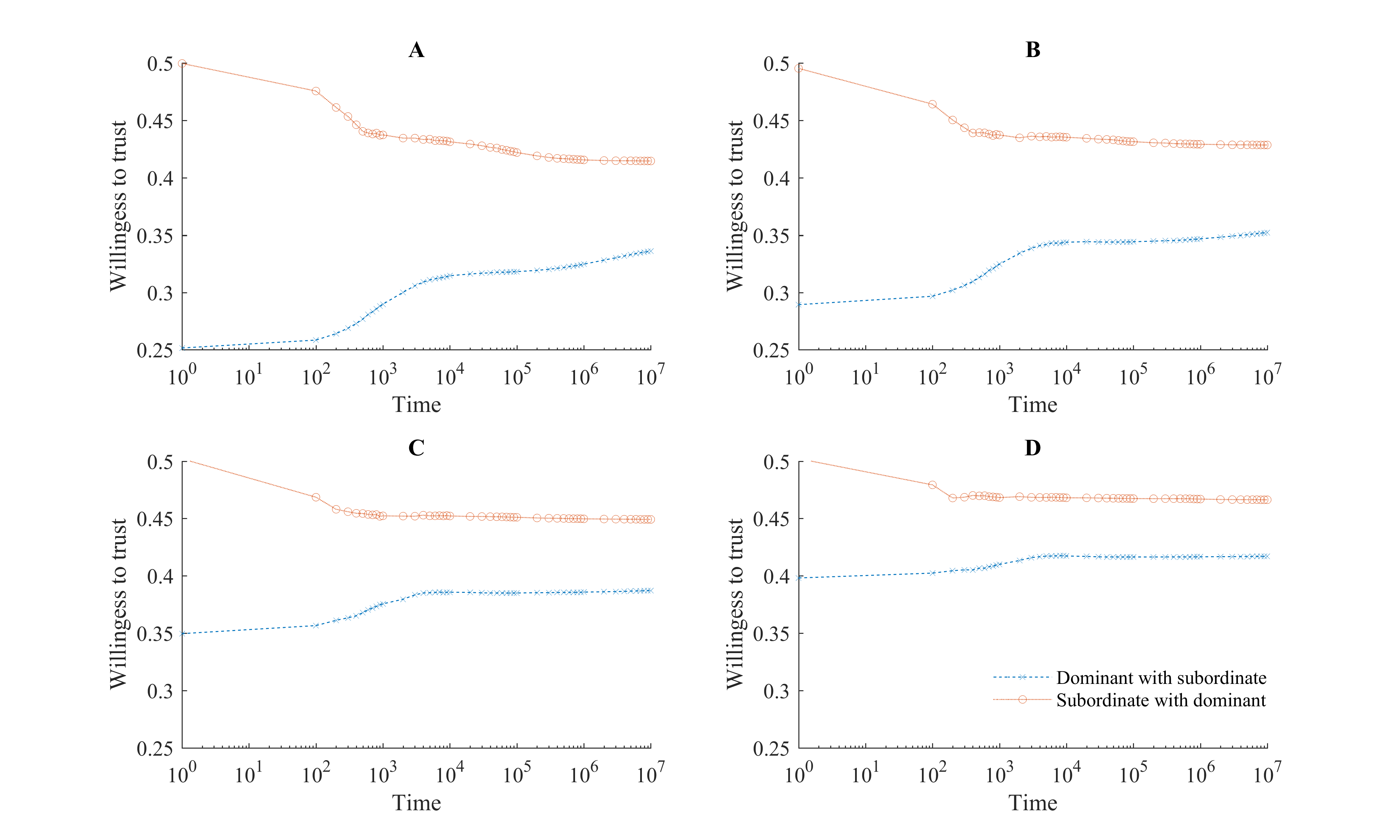

Supplement: S6 Fig — shows the dynamics of intergroup collaboration in a population where members initially tend to distrust each other. Specifically, we set ρj:I,0 ~ U(0,0.5) and ρi:J,0 ~ U(0,0.25). S6 Fig shows that low levels of trust in the population do not change the main qualitative observations. (TIF) [file pone.0194871.s008.tif]

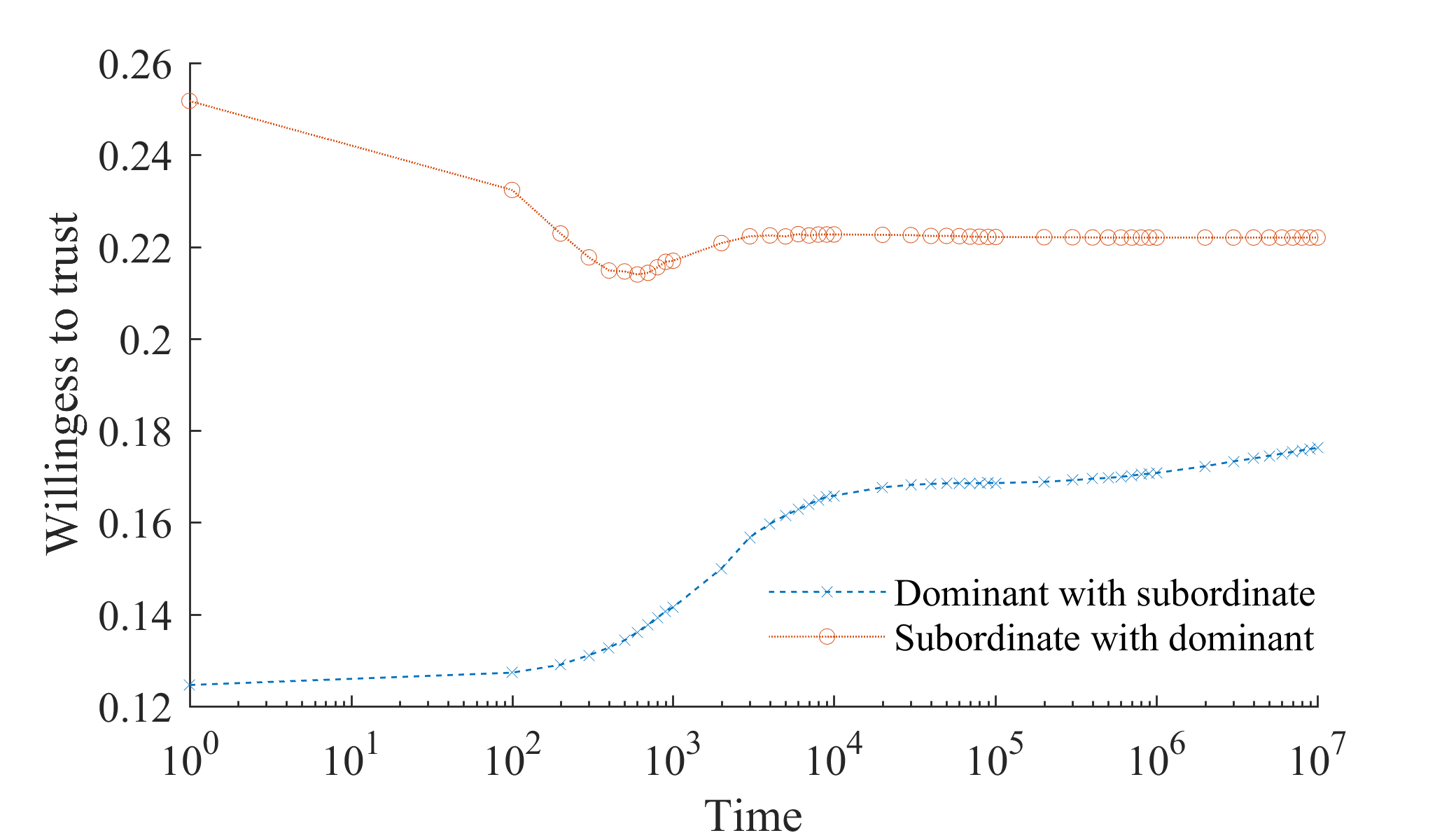

Supplement: S7 Fig — show the dynamics of willingness to trust when initial beliefs follow a truncated normal, and not uniform, distribution. Specifically, Panels A through D depict the dynamics of intergroup collaboration for the following initial distributions: A) ρj:I,0 ~ N(0.5,0.1) | ρj:I,0 ∈ (0,1) and ρi:J,0 ~ N(0.25,0.1) | ρj:I,0 ∈ (0,1), B) ρj:I,0 ~ N(0.5,0.2) | ρj:I,0 ∈ (0,1) and ρi:J,0 ~ N(0.25,0.2) | ρj:I,0 ∈ (0,1), C) ρj:I,0 ~ N(0.5,0.3) | ρj:I,0 ∈ (0,1) and ρi:J,0 ~ N(0.25,0.3) | ρj:I,0 ∈ (0,1), and D) ρj:I,0 ~ N(0.5,0.4) | ρj:I,0 ∈ (0,1) and ρi:J,0 ~ N(0.25,0.4) | ρj:I,0 ∈ (0,1). S7 Fig show that when the variance is large (D) the initial stigma is relatively small as the initial willingness to collaborate among dominant group members is comparatively high. This is an outcome of asymmetric truncation (mean = 0.25, distance to lower truncation = 0.25, distance to the upper truncation = 0.75). As the variance declines (D → A), stigma against the subordinate group becomes greater. Regardless of the level of variance, results of simulations with a truncated normal distribution of initial beliefs are consistent with our main observations (see Fig 4). (TIF) [file pone.0194871.s009.tif]

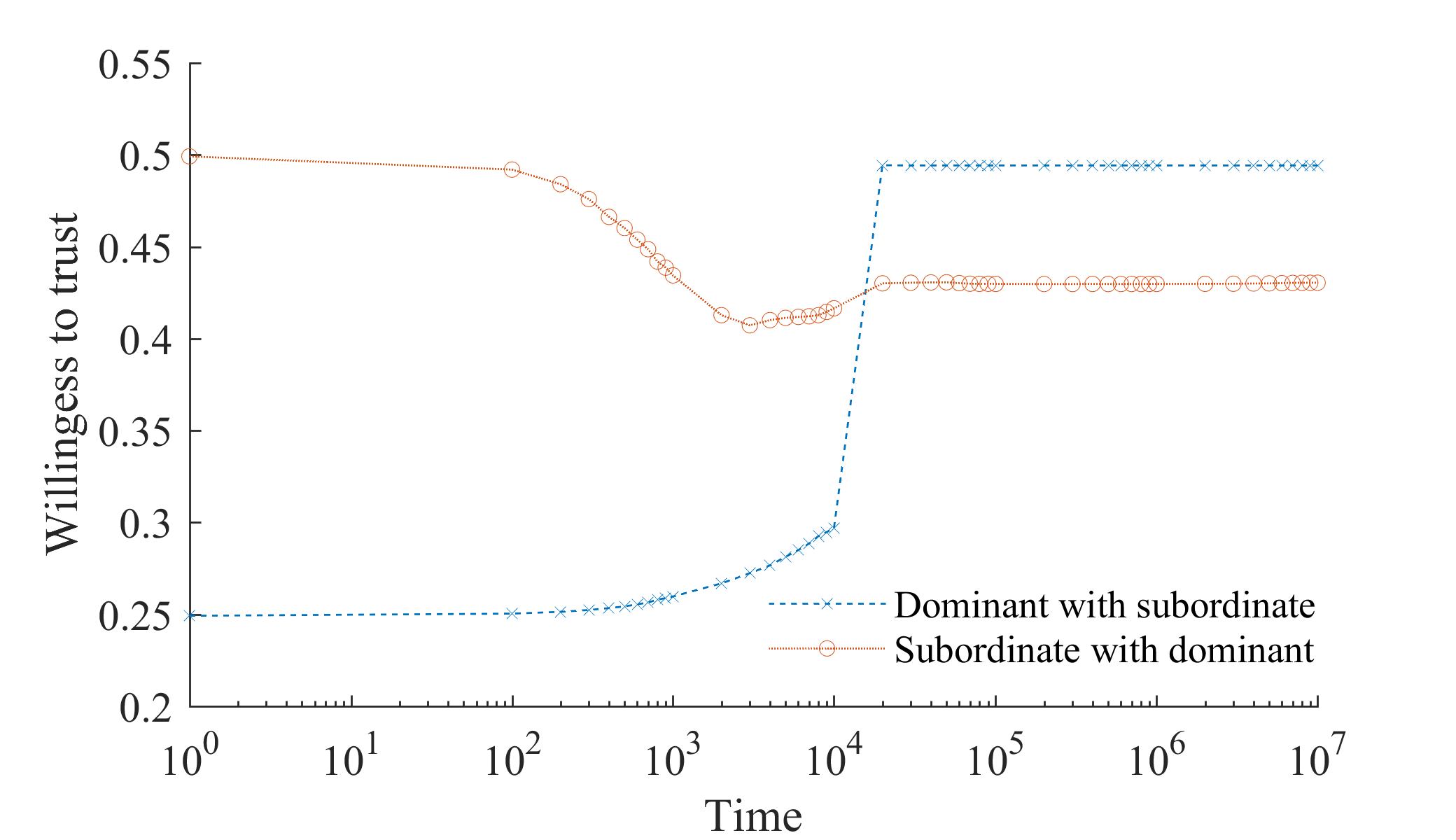

Supplement: S8 Fig — shows the consequences of perfect enlightenment at some distant moment in time. Specifically, we set enlightenment at t ≥ 105 (note that to preserve comparability with Fig 5 we keep the average count of accumulated interactions per individual constant at 102); beyond this moment all dominant group members do not see any difference between dominant and subordinate group members, i.e. do not discriminate. The main observation is that even perfect enlightenment, when put into action at such a late stage in societal development, cannot revive intergroup trust (cf. Fig 5). (TIF) [file pone.0194871.s010.tif]

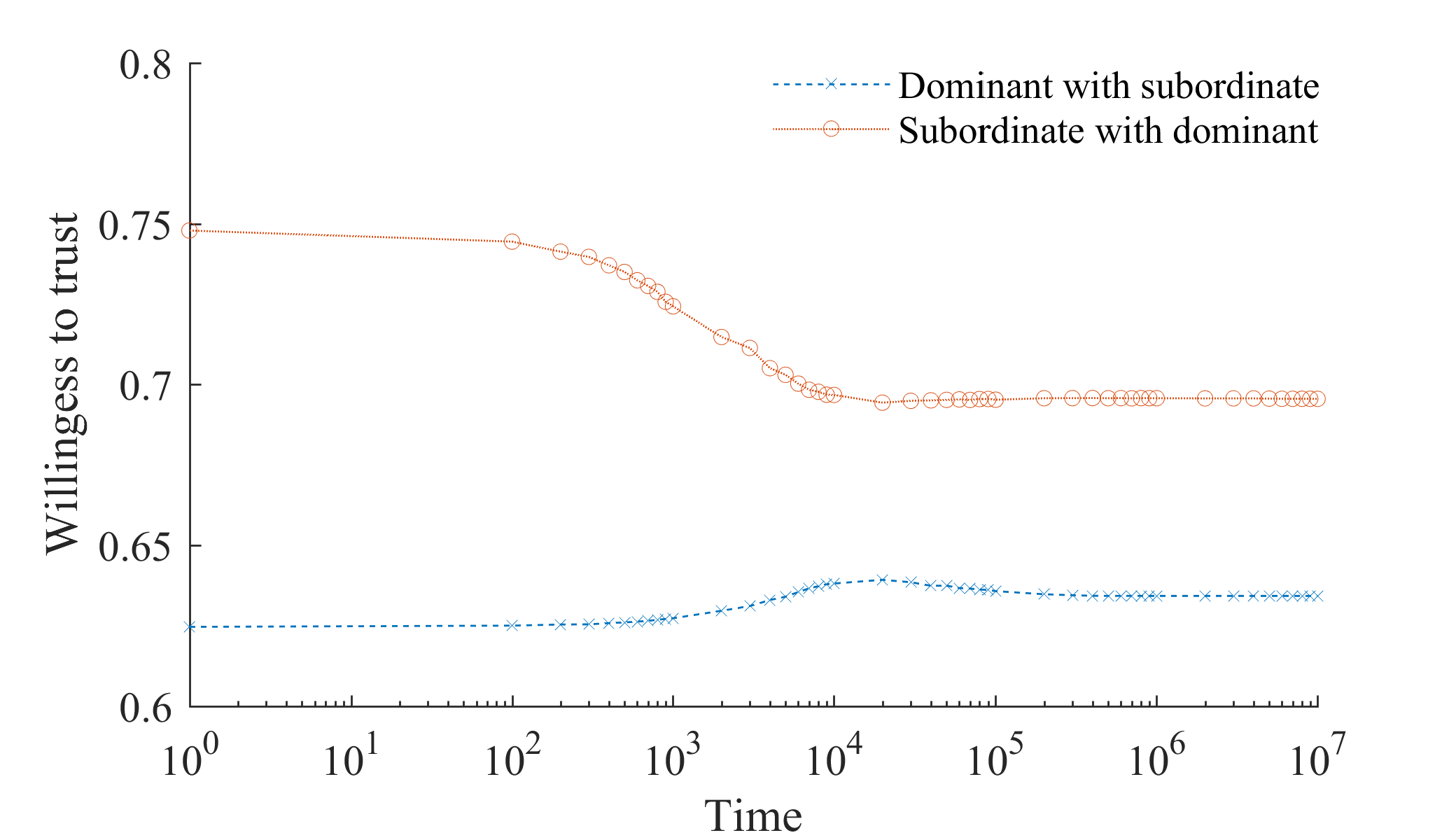

Supplement: S9 Fig — shows the dynamics of willingness to trust in a generally collaborative population. In this experiment we constrain individuals to collaborate with one another in social interactions. Specifically, we set ρj:I,0 ~ U(0.5,1) and ρi:J,0 ~ U(0.5,0.75). High levels of trust in a large population do not change the main qualitative observations. (TIF) [file pone.0194871.s011.tif]

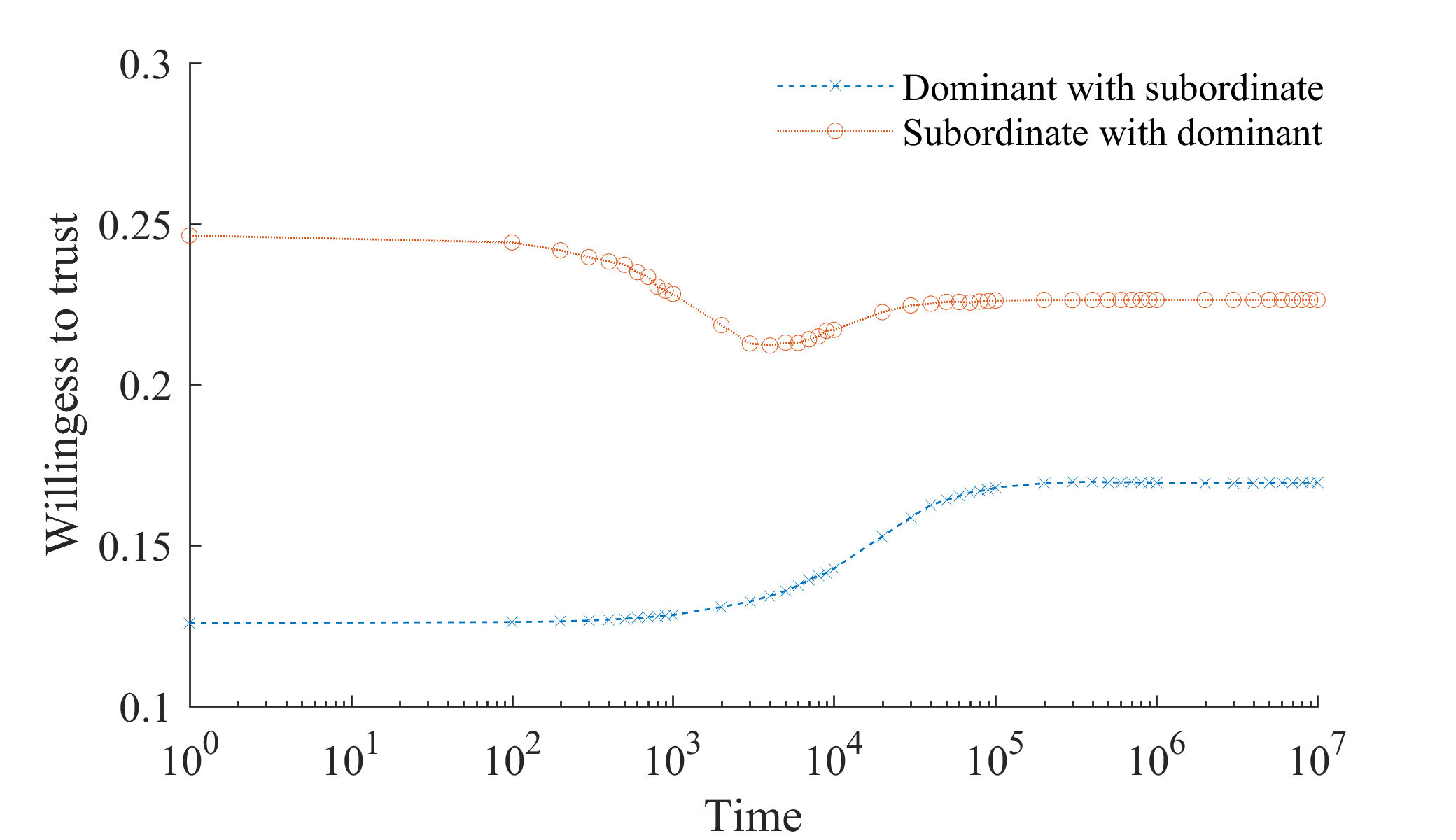

Supplement: S10 Fig — shows the dynamics of intergroup collaboration in a population where individuals initially tend to distrust each other. Specifically, we set ρj:I,0 ~ U(0,0.5) and ρi:J,0 ~ U(0,0.25). Low levels of trust in a large population do not change the main qualitative observations. (TIF) [file pone.0194871.s012.tif]

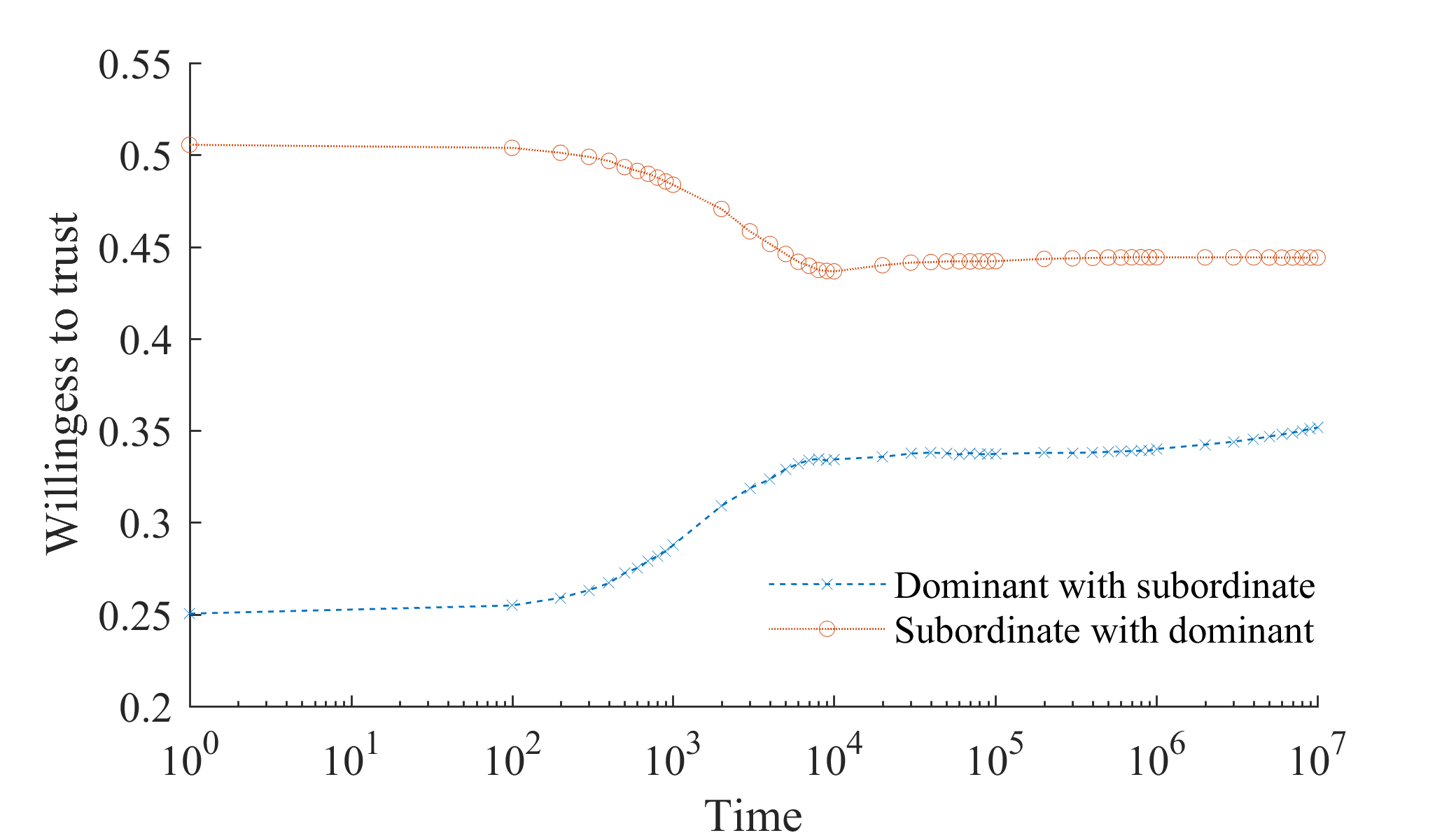

Supplement: S11 Fig — shows the dynamics of the willingness to cooperate in a society where the stigmatized subordinate group is actually numerically larger in size than the dominant group. Specifically, we assume that |I| = 200 and |J| = 800. Inversing the relative size of the groups in a large population has no qualitative effects on the persistence of stigma. (TIF) [file pone.0194871.s013.tif]

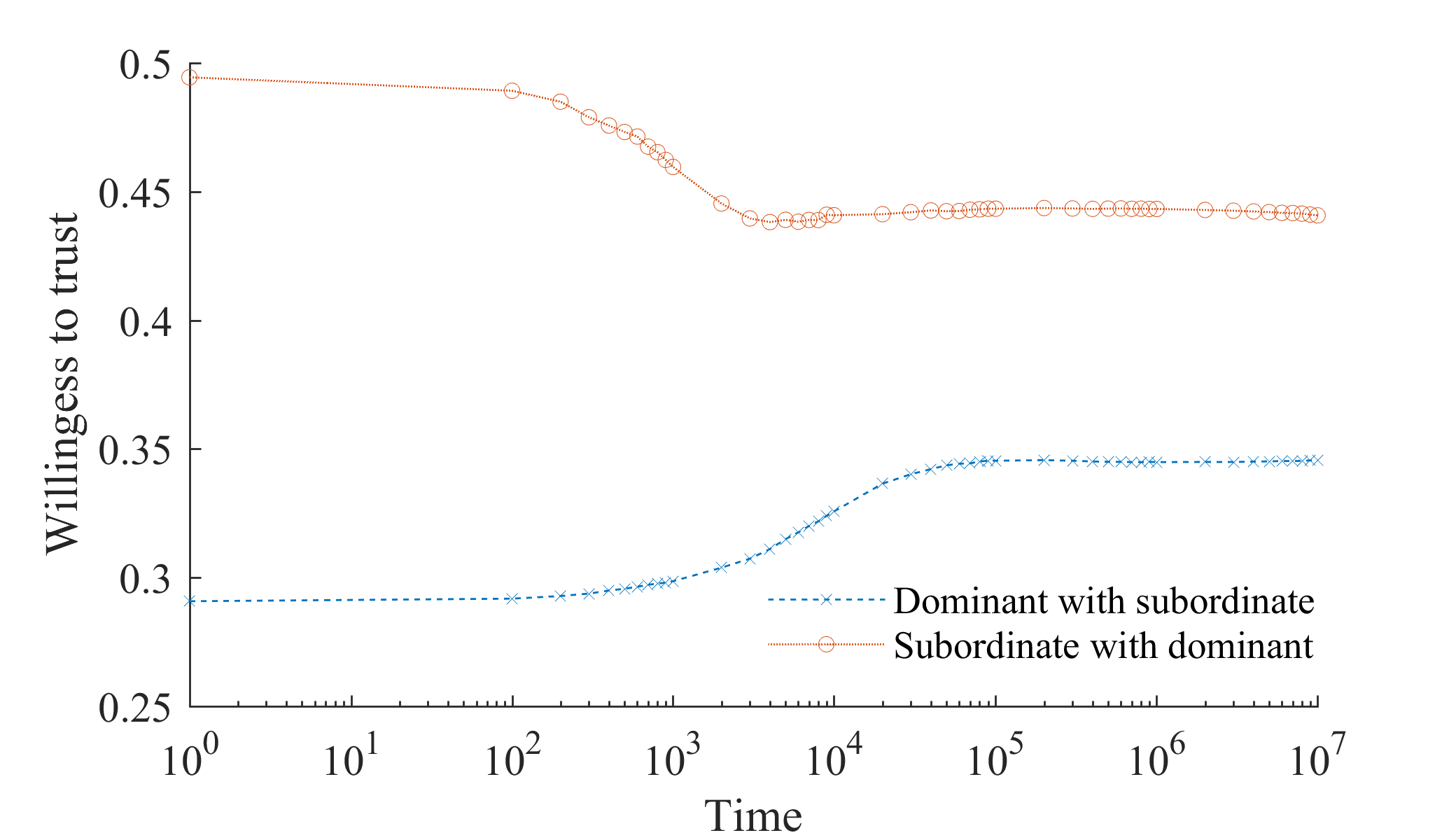

Supplement: S12 Fig — shows the dynamics of collaboration in a population where initial beliefs are distributed normally. Specifically, we set ρj:I,0 ~ N(0.5,0.2) | ρj:I,0 ∈ (0,1) and ρi:J,0 ~ N(0.25,0.2) | ρj:I,0 ∈ (0,1). As seen in S12 Fig, the main findings continue to hold. (TIF) [file pone.0194871.s014.tif]
